# Flyway‐Scale Assessment of Habitat Suitability and Key Environmental Drivers for Waterbirds in Southern China

**DOI:** 10.1002/ece3.72730

**Published:** 2025-12-23

**Authors:** Jiaxu Fan, Peng Du, Yi Lian, Lei Cui, Haixiao Li, Long He, Yuanyuan Tan, Xunqiang Mo, Zhengwang Zhang

**Affiliations:** ^1^ Faculty of Geographical Science Tianjin Normal University Tianjin China; ^2^ National Astronomical Observatory Chinese Academy of Sciences Beijing China; ^3^ Navigation College Jimei University Xiamen China; ^4^ School of Environment Science and Engineering Hubei Polytechnic University Wuhan China; ^5^ Ministry of Education Key Laboratory for Biodiversity Sciences and Ecological Engineering, College of Life Sciences Beijing Normal University Beijing China

**Keywords:** conservation gap, environmental factor, MaxEnt model, suitable habitats, waterbirds

## Abstract

Climate change and anthropogenic activities have caused widespread loss and degradation of waterbird habitats, posing a critical global conservation challenge. This study focuses on eastern and southern China, specifically the area south of the 800 mm isohyet, and examines 47 waterbird species. Using the MaxEnt species distribution model, we integrated multi‐source environmental variables—including climate, topography, vegetation, land use, and population density—to evaluate the habitat suitability of different ecological groups and identify spatial gaps in the current protection system. The results reveal a clear coastal‐to‐inland gradient in the distribution of suitable habitats, with high‐suitability hotspots concentrated in coastal regions and radiating inland along major river systems. Distinct ecological groups respond differently to environmental factors: NDVI and DEM were identified as key environmental factors for gulls and shorebird, which prefer low‐elevation, sparsely vegetated open habitats, while herons and large wading birds are more strongly associated with densely vegetated landscapes. Some groups, such as herons, show broader ecological adaptability, whereas shorebird tend to rely on more specialized habitat types. Spatial overlay analysis indicates a significant mismatch between existing nature reserves and high‐suitability areas, with particularly low protection coverage in southern and southwestern regions, highlighting the need for urgent ecological intervention. Our study reveals structural spatial deficiencies in current waterbird habitat conservation efforts and emphasizes the need for landscape‐scale conservation strategies, including wetland restoration, habitat corridor planning, and reserve network optimization, to improve the capacity of protected areas to support waterbird diversity and migratory connectivity. The findings provide valuable data support and spatial planning recommendations for future conservation management.

## Graphical abstract

This study assessed the distribution of suitable habitats and ecological drivers for 47 waterbird species in eastern and southern China (south of the 800 mm isohyet), revealing a clear coastal‐to‐inland gradient, with NDVI and DEM identified as key environmental variables. Different ecological groups exhibited distinct habitat preferences. The results also revealed a significant spatial mismatch between highly suitable areas and existing protected zones, particularly in rapidly urbanizing regions where protection coverage remains below 7%, highlighting the urgent need for landscape‐scale conservation strategies and improved reserve networks.

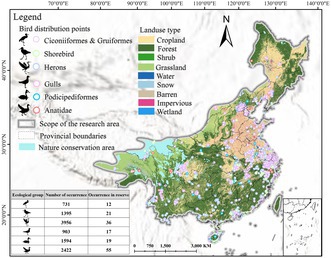

## 1 Introduction

Birds are widely used as bioindicators of ecosystem condition because they are well monitored, ecologically diverse, and responsive to environmental change (Qiu et al. 2024; Mekonen 2017). Within this group, waterbirds are especially informative for ecological monitoring and conservation because they combine long‐distance migratory connectivity with strong fidelity to wetland habitats, linking pressures across seasons to site‐level, measurable change (Panov et al. 2023; Amano et al. 2018; Green and Elmberg 2014). However, accelerating human activities and climate change are driving wetland loss, fragmentation, and degradation, which in turn threaten waterbird populations (Amano et al. 2020; Wang et al. 2022; de Felipe et al. 2024).

The East Asian–Australasian Flyway (EAAF) is a vital migratory route for over 50 million waterbirds each year, passing through China's coastal and riverine regions (Takekawa et al. 2023). From 2000 to 2015, China's natural coastal wetlands declined by approximately 10,000 km^2^, largely due to land reclamation and ecological degradation (Lin and Yu 2018). This habitat loss has contributed to observed declines in migratory waterbirds, particularly shorebird, along the EAAF, and it may continue if coastal pressures persist (Murray et al. 2019; Studds et al. 2017). Although waterbirds show some adaptive responses to environmental changes, these adaptive responses alone are insufficient to mitigate the long‐term impacts of climate change. Against this backdrop, understanding the spatial distribution, driving factors, and conservation gaps of waterbird habitats is crucial for effective biodiversity conservation and climate change mitigation.

A wealth of research has examined waterbird habitat distribution and their responses to environmental shifts, including habitat suitability modeling, climate‐driven habitat migration, and issues like habitat fragmentation and connectivity (Amano et al. 2020; Wang et al. 2022; Komori et al. 2023; Phillips et al. 2006; Gao et al. 2021). Species distribution models (SDMs), grounded in ecological niche theory, predict species' potential distribution and ecological suitability by integrating species data with environmental variables. They are essential for ecological conservation, habitat assessment, biodiversity protection, and predicting invasive species (Komori et al. 2023). The Maximum Entropy model (MaxEnt) is widely used because it performs robustly with presence‐only and small‐sample data while maintaining high predictive accuracy (Feng, Park, Walker, et al. 2019). For example, Huang et al. (2023) used MaxEnt to map suitable waterbird habitats around the Bohai Sea, identifying NDVI, soil cover, and maximum temperature as key factors driving habitat changes. Similar studies by Wen et al. (2016) and Sun et al. (2023) have highlighted the importance of identifying and protecting critical habitats, especially in coastal areas.

To date, most research has focused on small‐scale areas such as wetlands and protected zones. However, the reliance on artificial administrative boundaries limits our ability to capture habitat connectivity and broader, large‐scale trends. Using natural geographical divisions as boundaries offers a more accurate view of habitat continuity and large‐scale changes. The 800 mm precipitation line, which divides China's humid and semi‐humid regions, is both an ecological boundary and an economic indicator (Yuan et al. 2014). Areas south and east of this line are vital parts of the EAAF, hosting key wintering and stopover sites for waterbirds. However, high population density and intense economic activity in this region lead to land‐use pressures, habitat fragmentation, and degradation for waterbirds (Zhang et al. 2024). The overlap of ecological and economic factors makes this area crucial for balancing biodiversity conservation with economic development.

This study focuses on the region south and east of the 800 mm precipitation line in China, considering the effects of climate, land use, and topography on waterbirds and their habitats. Using the MaxEnt model, we evaluate habitat suitability for 47 waterbird species to analyze habitat distribution and driving mechanisms. The main objectives are: (1) to identify habitat distribution patterns and environmental driving factors; (2) to assess the combined impact of human activities and landscape patterns on habitat distribution; and (3) to identify gaps in the conservation network and offer recommendations for optimizing regional strategies.

## 2 Methods and Materials

### 2.1 Study Area

**FIGURE 1:**
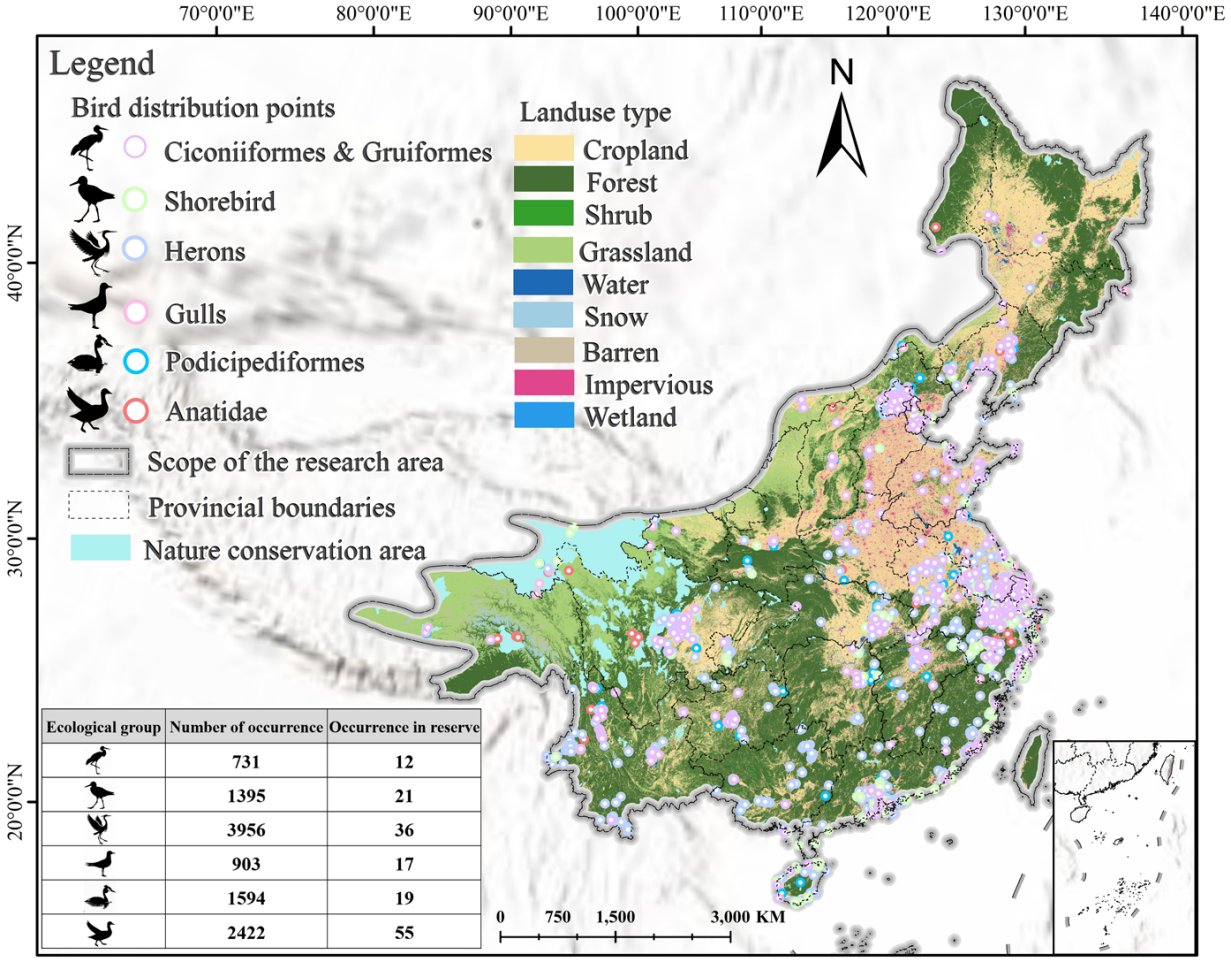
Nature conservation area, bird distribution points, and land‐use types in the study area. Numbers in the table denote counts of waterbird occurrence records within the study area during 2022 (not species). “Occurrence in reserve” indicates records whose coordinates fall inside protected‐area boundaries. The bottom‐right inset shows the South China Sea Islands.

This study focuses on the region south and east of the 800 mm precipitation line in China, covering approximately 5.4 million km^2^ across northern, southern, and parts of central provinces (Figure 1). The 800 mm isohyet itself represents an important ecological boundary dividing humid and semi‐humid climatic zones, while the area to its south and east corresponds to some of the most densely populated and economically developed regions of the country (Qin et al. 2013). Characterized by high precipitation and diverse ecosystems—including lakes, rivers, coastal wetlands, and rice‐paddy mosaics—this region provides key habitats for waterbirds to breed, overwinter, and stop over during migration (Lei et al. 2019). As a core segment of the EAAF, it plays an irreplaceable role in sustaining global waterbirds' diversity (Takekawa et al. 2023).

### 2.2 Data Selection

#### 2.2.1 Bird Distribution Data

This study utilized GBIF citizen science data (https:// doi.org/10.15468/dl.dbet8a) to obtain bird distribution records in China for 2022, totaling 388,924 records across 1205 species, covering 80.01% of the known avian species in the country (Zheng 2023a, 2023b), including 201 species of waterbirds. With broad spatial and temporal coverage, these data provide a robust basis for assessing avian diversity and environmental responses. Recognizing potential differences in observation standards and reporting patterns in citizen‐science compilations, species names and higher classifications were manually verified against authoritative avian taxonomies, and miscoded records (e.g., misspellings, taxonomic misassignments) were removed. To address uneven spatial distribution of observer effort, potential sampling biases in GBIF data and to align occurrences with the 1‐km^2^ resolution of environmental predictors, 1‐km^2^ grid de‐duplication was applied, retaining a single record per species per grid cell. This procedure reduces the relative influence of highly sampled localities while preserving broad‐scale distribution signals relevant to the analysis (Boria et al. 2014). Residual structure in reporting effort may remain—particularly for species represented by few occurrences—so interpretation emphasizes large‐scale suitability patterns rather than fine‐scale, site‐level predictions (Roberts et al. 2017).

Following the classification framework of Chinese waterbirds (Liu et al. 2019), we selected representative species from six ecological groups for analysis. These included three types of waders (Ciconiiformes and Gruiformes, Shorebird, Herons) and three types of waterfowl (Gulls, Podicipediformes, Anatidae) (Table 1). In this study, the ecological taxa are used as functional ecological categories rather than strict taxonomic units, aiming to reflect the similarities among species in habitat use, foraging behavior, and ecological roles. Therefore, some groups may include species from different families or orders that share comparable ecological niches, which helps provide a more integrated interpretation of habitat patterns. On this basis, we organized and screened the species occurrence data. After rigorous quality control, we retained 11,001 valid occurrence records for 47 waterbird species (Figure 1). The number of occurrence records for each species is detailed in Table S1.

**TABLE 1:** Ecological taxonomic classification of selected bird species.

Ecological taxa	Species
Ciconiiformes & Gruiformes	*Ciconia boyciana, Grus grus, G. vipio, Fulica atra*
Shorebird	*Calidris acuminata, C* *. alpina* , *C. canutus, C* *. tenuirostris* , *C* *. ruficollis* , *C* *. ferruginea* , *Charadrius mongolus, C. alexandrinus, Himantopus himantopus, Tringa stagnatilis, T. nebularia, Recurvirostra avosetta, Pluvialis squatarola, Numenius arquata, Limosa limosa*
Herons	*Platalea leucorodia, Nycticorax nycticorax, Egretta garzetta, Ardeola bacchus, Ardea cinerea*
Gulls	*Sternula albifrons, Sterna hirundo, Larus crassirostris, L* *. vegae* , *Ichthyaetus relictus, Chroicocephalus ridibundus, Chlidonias hybrida*
Podicipediformes	*Tachybaptus ruficollis, Podiceps cristatus*
Anatidae	*Anas zonorhyncha, A* *. acuta* , *Anser. fabalis, A* *. anser* , *Aythya ferina, Bucephala clangula, Cygnus cygnus, C* *. columbianus* , *Mareca falcata, M. penelope, M. strepera, Mergellus albellus, Mergus merganser, Tadorna tadorna*

#### 2.2.2 Environmental Factor Data

This study selected 25 environmental variables based on ecological principles (Anderle et al. 2022), categorized into four major groups: climate, topography and vegetation, land‐use, and human activity (Table 2). All datasets were obtained from authoritative scientific sources and were resampled to a uniform spatial resolution to ensure consistency and comparability among variables. Table S2 provides the full details for every dataset and variable, ensuring transparency and reproducibility.

**TABLE 2:** Environmental Factors and Their Categories.

Category	Name remarks	Variable	Unit	Data type
Climate factors	Bio1	Annual mean temperature	°C	Continuous
	Bio2	Mean diurnal range	°C	Continuous
	Bio3	Isothermality	Dimensionless	Continuous
	Bio4	Temperature seasonality	Dimensionless	Continuous
	Bio5	Max temperature of warmest month	°C	Continuous
	Bio6	Min temperature of coldest month	°C	Continuous
	Bio7	Temperature annual range	°C	Continuous
	Bio8	Mean temperature of wettest quarter	°C	Continuous
	Bio9	Mean temperature of driest quarter	°C	Continuous
	Bio10	Mean temperature of warmest quarter	°C	Continuous
	Bio11	Mean temperature of coldest quarter	°C	Continuous
	Bio12	Annual precipitation	mm	Continuous
	Bio13	Precipitation of warmest month	mm	Continuous
	Bio14	Precipitation of driest month	mm	Continuous
	Bio15	Precipitation seasonality	Dimensionless	Continuous
	Bio16	Precipitation of wettest quarter	mm	Continuous
	Bio17	Precipitation of driest quarte	mm	Continuous
	Bio18	Precipitation of warmest quarter	mm	Continuous
	Bio19	Precipitation of coldest quarter	mm	Continuous
Topographic and vegetation factors	DEM	Digital elevation model	m	Continuous
	SLO	Slope	°	Continuous
	ASP	Aspect	°	Continuous
	NDVI	Normalized difference vegetation index	Dimensionless	Continuous
Land‐use factor	LC	Type of land use	N/A	Classification
Human disturbance factor	POP	Population density	/Km^2^	Continuous

Climate data were derived from the World Climate Database (Fick and Hijmans 2017) and integrated with latitude, longitude, and elevation using spline interpolation. Topographical data are obtained from the Copernicus PANDA platform's DEM (Airbus 2020), from which slope and aspect parameters are extracted using ArcGIS. Vegetation data are represented by the NDVI index from the National Ecosystem Science Data Center (Yang et al. 2019). Land use data are based on the 30‐m resolution annual land cover dataset released by the Wuhan University team (Yang and Huang 2023). Human activity indicators are constructed by extracting impervious surface information from land cover data and combining it with the 2022 provincial population statistics (National Bureau of Statistics of China 2023) to generate a 300‐m resolution population density raster. The environmental variables used in this study come from datasets with different reference periods, and a mixed‐temporal framework was adopted to balance ecological relevance, data availability, and interpretability. Topographic factors such as elevation were regarded as essentially invariant; vegetation and climate factors were represented by long‐term averages to capture stable gradients in temperature, precipitation, and vegetation structure; and land use and human disturbance factors were anchored as closely as possible to 2022 to reflect contemporary human pressure. This approach reduces the influence of single‐year anomalies on the models and enhances the robustness and comparability of the conclusions across years and regions.

All datasets were resampled to a uniform spatial resolution of 300 × 300 m to harmonize multi‐source layers originally available at finer and coarser scales, striking a balance between computational feasibility and preserving sub‐kilometer habitat heterogeneity. We applied the nearest‐neighbor method during resampling to minimize distortions (Congalton and Green 2019), and standardized to the WGS_1984_UTM_48N coordinate system to ensure a consistent spatial reference for modeling. This indicator system comprehensively reflects the ecological characteristics of waterbird habitats and human disturbance factors, providing an integrated variable framework consistent with the analytical objectives of modeling species–environment relationships and identifying key drivers of habitat suitability.

Nighttime light (NET) data were used to represent human activity intensity and its ecological pressure on waterbird habitat suitability, and spatial conservation gaps were identified by overlaying modeled suitable habitats with the national conservation‐areas layer. Both datasets were sourced from the Resource and Environmental Science Data Platform (Xu 2022).

The selection of environmental variables is essential for ensuring model accuracy, as excessive variables can introduce multicollinearity, leading to overfitting and reduced reliability (Ma and Sun 2018; He et al. 2021). In this study, all variables (Table 2) were initially screened using a two‐step procedure: (i) contribution screening and removal of zero‐contribution predictors, and (ii) correlation filtering based on Pearson coefficients at species occurrence points. The contribution of each variable was evaluated using the built‐in jackknife test in MaxEnt, which quantifies a predictor's independent explanatory power by measuring its effect on model gain (Phillips et al. 2006). For each species, among variable pairs with |r| ≥ 0.8, the predictor with the lower contribution was removed. The remaining variables were used in the final model (Table S3). This procedure selects optimal predictors for each species while minimizing multicollinearity and improving the reliability of habitat‐suitability predictions.

### 2.3 Methodology

#### 2.3.1 Workflow of the Methods

**FIGURE 2:**
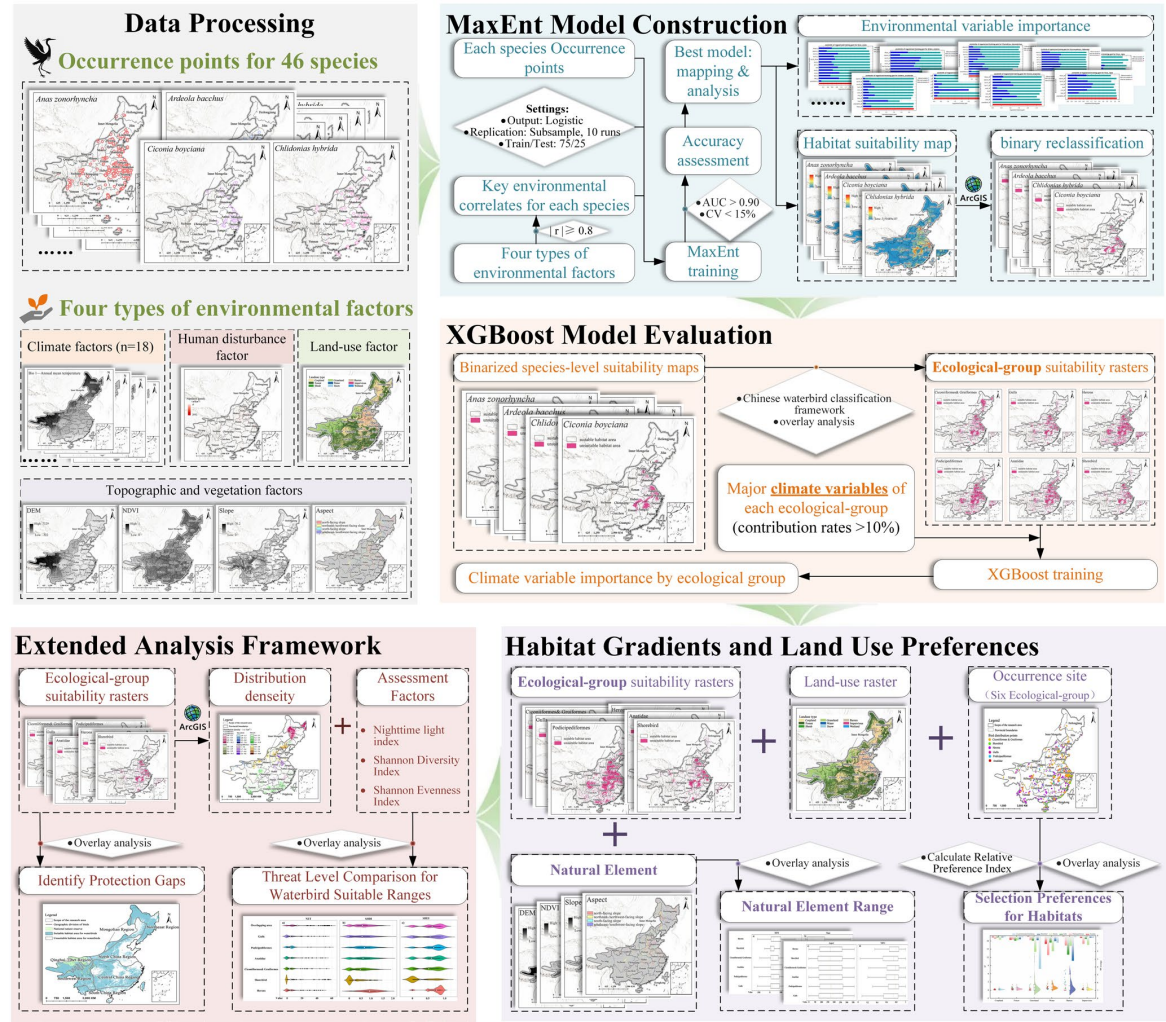
Workflow of the Methods.

This study establishes an integrated analytical framework that combines waterbird occurrence records with multiple categories of environmental variables, aiming to systematically model habitat suitability, identify environmental drivers across scales, and ultimately provide spatially explicit scientific evidence for conservation prioritization (Figure 2). First, to accurately capture the distinct ecological niche requirements of different waterbird species, the MaxEnt model was employed to construct customized distribution models for 46 species. Through a pre‐screening process, environmental variables were filtered to ensure that each model achieved an optimal balance between ecological interpretability and predictive performance, resulting in reliable binarized habitat suitability maps that serve as the foundation for subsequent analyses. Subsequently, given that species‐level analyses, although detailed, tend to be fragmented and thus insufficient to reveal overarching ecological patterns or inform coherent conservation strategies, the analytical scale was elevated to the ecological‐group level. By overlaying individual species distribution maps, composite habitat maps were generated for each group, and the XGBoost model was applied to objectively quantify the relative importance of climatic factors shaping each group's distribution pattern. Building upon the established climate‐driven macro‐distribution framework, the study then focused on functional habitat preferences of different ecological groups, characterizing fine‐scale habitat structures by calculating relative preference indices for land‐use types and analyzing the distributional features of topographic and vegetation variables. Finally, to bridge scientific understanding with conservation practice, all suitable habitats of waterbirds were integrated to assess risks along anthropogenic pressure gradients and systematically compared with existing protected areas under the biogeographic regionalization framework of G. Zheng (2012). This process enabled the precise identification of critical conservation gaps. Overall, the workflow follows a progressive logic from fundamental distribution modeling, through mechanism‐based analysis, to conservation‐oriented applications—forming a coherent technical pathway that links ecological understanding to spatial conservation decision‐making.

#### 2.3.2 MaxEnt Model Construction

The MaxEnt model relies on the key assumption that occurrence records represent an unbiased sample from the species’ environmental distribution. The MaxEnt model was configured with the output format set to “Logistic”, file type as “ASC”, and replication type as “Subsample”. For each species, 10,000 background points were sampled to represent the environmental background across the study area. We enabled ‘Create response curves’ and ‘Jackknife tests for variable importance’ to assess variable contributions and marginal effects. To evaluate model performance and mitigate overfitting, we implemented a subsampling cross‐validation approach, using 75% of occurrence records for training and 25% for testing (Gao et al. 2023). Each model was run 10 times, and the average predicted suitability was used as the final habitat suitability map, exported in ASC format. Model accuracy was evaluated using the Area Under the Receiver Operating Characteristic Curve (AUC) (Ma and Sun 2018), where values above 0.9 indicate high reliability (He et al. 2021). Model stability was assessed using the coefficient of variation (CV) across the 10 replicates, with AUC > 0.90 and CV < 15% considered as thresholds for reliable and stable model performance (He et al. 2021).

Analyzing habitat suitability at the ecological‐group level allows species‐specific differences to be integrated into broader functional patterns, providing a coherent framework to understand community‐level habitat use and to identify key climatic and ecological influences that guide conservation priorities for both common and threatened species (Mayani‐Parás et al. 2023; Sohil and Sharma 2020). To identify the suitable habitat range and hotspot distribution for waterbird groups, the model prediction results were imported into ArcGIS for raster reclassification. After generating binary habitat suitability maps for each species, the maps were overlaid. The natural breaks classification method, which best reflects the actual distribution patterns (Javidan et al. 2021), was applied to reclassify the overall habitat suitability into three categories: non‐suitable, low‐suitability, and high‐suitability. High‐suitability areas were defined as suitable habitats, while the others were considered unsuitable. This reclassification produced binary habitat maps for each species, with the corresponding suitability thresholds summarized in Table S1.

In this study, we regarded environmental variables with a percent contribution below 10% in the MaxEnt model as non‐primary driving factors and excluded them from subsequent key factor analyses. On one hand, this threshold was based on empirical practices in previous species distribution modeling studies, where 5% or 10% is commonly used to reduce model complexity and focus on ecologically meaningful variables (Liu et al. 2022; Sun et al. 2022). On the other hand, the percent contribution in MaxEnt reflects the improvement in model gain attributed to each variable during iterations. Although this metric may be biased in the presence of multicollinearity, when correlations among variables are low, a contribution above 10% generally indicates ecological relevance in species niche formation (Phillips et al. 2006). Therefore, to ensure model interpretability and simplicity while avoiding redundancy from low‐contribution variables, we adopted 10% as the threshold for identifying core environmental variables with stable ecological significance across species models.

#### 2.3.3 XGBoost Modeling of Climatic Factors

Systematically identifying and quantifying the roles of climatic variables is a critical step in understanding and predicting the distribution and changing trends of waterbird habitats (Gaget et al. 2022; Steen and Powell 2012). Since the MaxEnt model was constructed separately for each species and included a multicollinearity filtering process for climatic variables (Bio1–Bio19) prior to modeling, the resulting variable importance values are species‐specific. Consequently, to examine climatic influences in a consistent and comparable manner across ecological groups, we further introduced the XGBoost (eXtreme Gradient Boosting) algorithm as a supplementary classifier.

Compared with traditional algorithms such as logistic regression and random forest, XGBoost offers significant advantages in model generalization, training efficiency, and robustness of feature importance assessment (Chen and Guestrin 2016), and has been widely applied in biodiversity conservation studies (Zhai et al. 2024; Miller et al. 2023; Valavi et al. 2022; Cha et al. 2021). In this study, we adopted a regularized gradient‐boosting configuration with a binary logistic objective, shallow additive trees, learning‐rate shrinkage, stochastic subsampling, and early stopping on a held‐out validation split to capture non‐linear interactions while preventing overfitting (Chen and Guestrin 2016).

In the modeling process, we used the binary habitat suitability of each ecological group (suitable or unsuitable) as the response variable and included the major climatic variables (contribution > 10%) identified in species‐level MaxEnt models as explanatory variables, enabling a unified quantification of the influence of climate factors on habitat suitability across ecological groups. The dataset was randomly divided into 70% for training and 30% for testing. Because the numbers of suitable and unsuitable pixels were imbalanced, class weighting was used to improve estimation under skewed class distributions (He and Garcia 2009). Hyperparameters that control tree complexity and regularization were selected by fivefold stratified cross‐validation conducted within the training partition to avoid information leakage (Varma and Simon 2006), with selection favoring configurations that maximized mean Overall Accuracy and minimized variability across folds (Roberts et al. 2017); when performances were comparable, simpler models were preferred to enhance generalization. Using the same cross‐validation folds, we summarized variable‐importance scores by their fold means and standard deviations to assess stability (Elith et al. 2008). Model performance was reported by Overall Accuracy, and variable importance by the Gain metric, defined as the average improvement in split quality attributable to each predictor (Chen and Guestrin 2016).

#### 2.3.4 Habitat Gradients and Land Use Preferences

Beyond macro‐scale climatic modeling, we incorporated additional analyses of micro‐topographic, vegetative, and land‐use preferences. Specifically, we extracted pixel‐level values of DEM, Slope, Aspect, and NDVI from the binary habitat suitability layers predicted by MaxEnt. These variables are closely related to hydrological and vegetation processes and have been shown to effectively characterize habitat quality in previous studies on migratory waterbirds (Gao et al. 2021; Henriques et al. 2022). For each ecological group, the median and interquartile range of these variables were calculated and visualized using box plots to depict ecological amplitude and distribution overlap across local environmental gradients.

To assess waterbirds' preferences for different land use types, we referred to previous studies based on the use‐availability framework (Santos et al. 2023; Boggie et al. 2018) and developed a Relative Preference Index (RF) based on MaxEnt model outputs. The index is defined as follows:

$${\mathrm{RF}}_{i,l}=\frac{U_{i,l}}{S_{i,l}}\times 10^{4}$$

where i denotes the ecological group, l represents the land use type, $U_{i,l}$ is the total area of observed distribution pixels within land type l for group i, and $S_{i,l}$ is the total area of MaxEnt‐predicted suitable habitat within the same land type. Given the limited number of bird occurrence points and substantial variation in land type coverage across the study area, the original RF values were often small and difficult to interpret. To enhance visual clarity and comparative resolution, all RF values were uniformly scaled by a factor of 10^4^. This scale transformation does not alter the relative differences among ecological groups (Fritsch et al. 2020). The RF index was calculated within the spatial extent of MaxEnt‐predicted suitable areas for each ecological group, allowing the evaluation of land‐use associations within a group‐specific suitability context. This approach partially accounts for differences in land‐cover area among habitat types and improves the comparability of land‐use patterns across ecological groups. Although the MaxEnt models balanced differences in sample size through feature selection and regularization, the spatial distribution of occurrence records may still be affected by detectability and sampling accessibility. Therefore, the RF values can be regarded as relative quantitative indicators of land‐use association, reflecting the relative level of habitat use among land‐cover types rather than absolute preference values.

#### 2.3.5 Extended Analysis Framework for Habitat Suitability Results

Considering that the core modeling mechanism of MaxEnt is more suitable for characterizing species' ecological niche responses to long‐term stable environmental gradients, it is limited in representing dynamic environmental variables influenced by anthropogenic disturbance, land use change, or landscape structure (Feng, Park, Liang, et al. 2019). Therefore, in this study, macro‐scale ecological variables such as climate, topography, and vegetation were selected during the modeling stage to emphasize the simulation of dominant niche‐defining factors, while avoiding the interpretative bias and reduced generalizability that may result from directly incorporating variables with high spatial heterogeneity and classification dependency (Suárez‐Seoane et al. 2020). Landscape structure indicators (e.g., SHDI, SHEI) and anthropogenic disturbance variables (e.g., nighttime light intensity) typically operate at meso‐ or micro‐scales, and their ecological effects depend on finer‐scale spatial configurations, land use structures, and disturbance intensity, which are difficult to accurately quantify across the large spatial extent (> 5 million km^2^) used in this study (Gao et al. 2021; Boggie et al. 2018). In addition, such variables are highly sensitive to land cover classification schemes and the size of analysis windows, lacking the standardization and cross‐regional comparability of climatic variables, and may reduce model robustness and transferability when directly included (Suárez‐Seoane et al. 2020).

This study constructed an independent ecological interpretation framework to extend the analysis beyond the predictive modeling stage. The framework aimed to interpret habitat suitability results from the perspective of landscape configuration and anthropogenic disturbance, allowing spatially explicit ecological inference without altering the MaxEnt model itself. Landscape metrics were quantified based on 30 m resolution land‐use data to quantify local‐scale heterogeneity around suitable habitats. Following Wang et al. (2011), a moving window approach was adopted with a 900 × 900 m window to capture the variation in landscape composition and configuration surrounding each pixel, enabling the assessment of habitat structure at an ecologically meaningful scale. Two landscape‐level indices—Shannon's Diversity Index (SHDI) and Shannon's Evenness Index (SHEI)—were then calculated to represent landscape diversity and compositional evenness, respectively. In addition, the Nighttime Light Index (NET) was introduced as a supplementary variable to characterize anthropogenic intensity and spatial disturbance gradients associated with waterbird habitats (Horton et al. 2023).

As part of the extended analysis framework, this study further evaluated the spatial congruence between predicted suitable habitats and existing protected areas to assess the spatial effectiveness of the current conservation network. Specifically, the MaxEnt‐predicted waterbird habitat layers were overlaid with the distribution of national nature reserves to identify regions of insufficient or mismatched protection (Tian et al. 2023). To enhance the biogeographic relevance of spatial statistics, the study aggregated suitability and protection data based on the bird biogeographic divisions proposed by Zheng Guangmei (Zheng 2023a, 2023b). For each division, we calculated key indicators including suitable habitat area, protected area size, their overlap, and protection coverage ratio. This method provides a more ecologically representative perspective for evaluating conservation effectiveness and offers scientific support for identifying protection gaps and optimizing strategies for reserve expansion and critical habitat management (Cai et al. 2024).

## 3 Result

### 3.1 MaxEnt Model Accuracy

**FIGURE 3:**
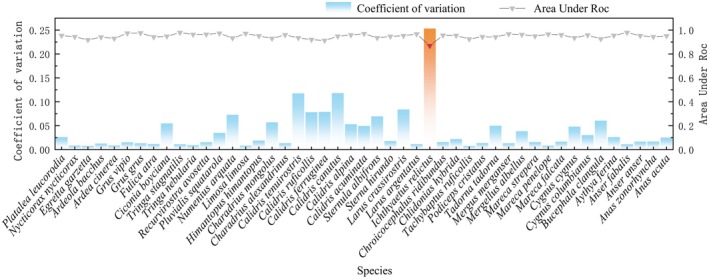
Mean AUC (Area Under the Curve, dimensionless) and coefficient of variation for 10 iterations of the MaxEnt model.

The habitat suitability prediction results (Figure S1–S6) show that, except for *Ichthyaetus relictus* , the average AUC values for the remaining species all exceed 0.90, and the stability metric CV is below 15% (Figure 3), indicating that the habitat suitability simulations exhibit high accuracy and stability. Therefore, the prediction result for *Ichthyaetus relictus* was excluded, and only the remaining 46 species were retained for subsequent analysis.

### 3.2 Spatial Distribution Patterns of Suitable Habitats

**FIGURE 4:**
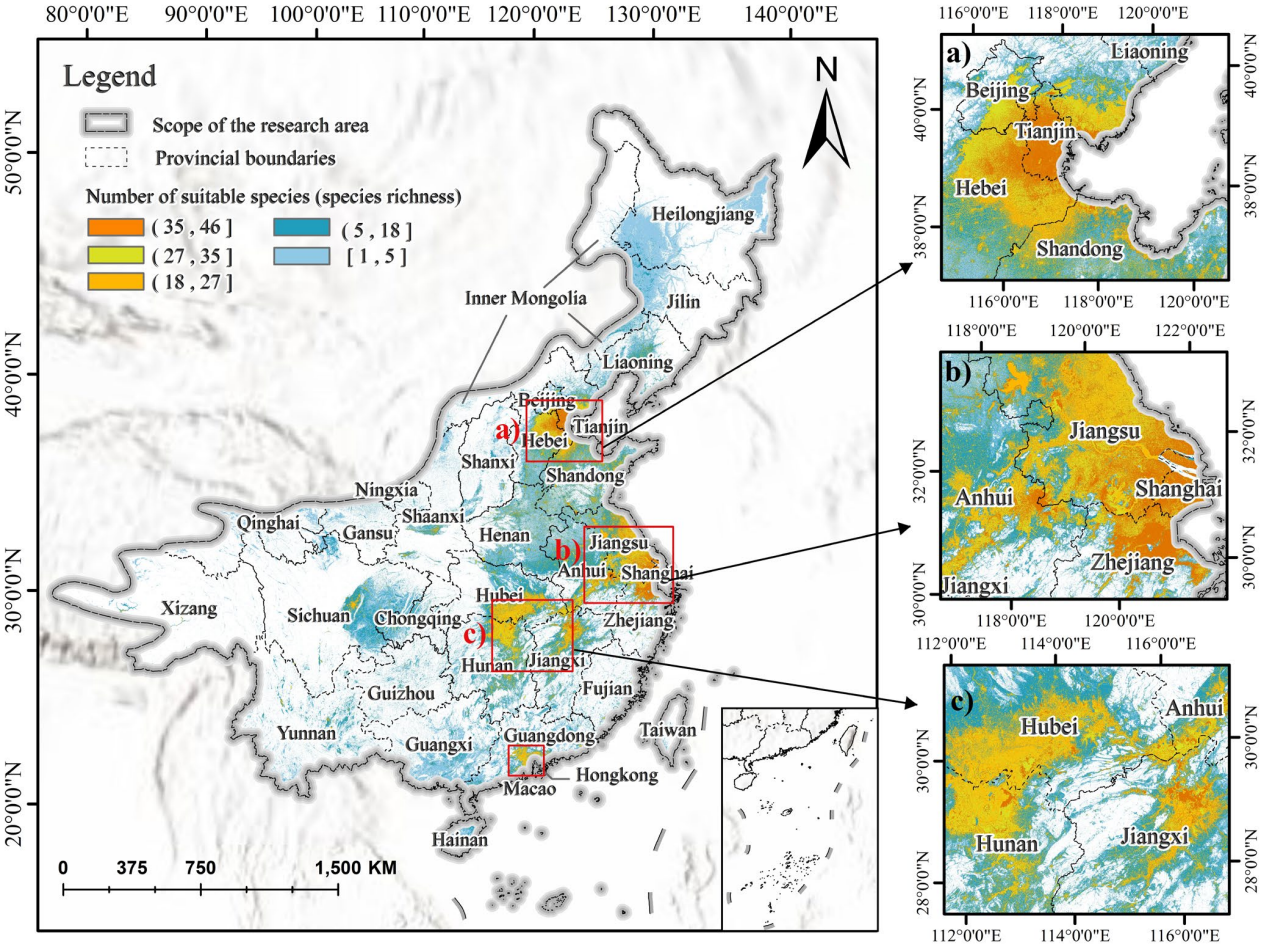
Spatial distribution of hotspot areas suitable for waterbirds. The map was generated by overlaying the binary habitat suitability rasters of all modeled species. Warmer colors indicate higher species richness and greater habitat suitability. Insets show regional hotspot clusters: (a) North China Plain (Beijing–Tianjin–Hebei–Shandong region); (b) Yangtze River Delta (Jiangsu–Shanghai–Zhejiang–Anhui region); (c) Middle Yangtze Basin (Hubei–Hunan–Jiangxi region).

The waterbird habitat suitability hotspots were derived by overlaying the binary suitability rasters of all modeled species (Figure 4). The results indicate that coastal regions exhibit markedly higher habitat suitability than inland areas, with major hotspots concentrated around coastal cities such as Tianjin and Shanghai. Inland suitable habitats are primarily distributed along major river systems in Hubei, Hunan, Jiangxi, and Anhui provinces. Several smaller suitable patches are also distributed along the eastern and central coast of Guangdong Province, with a key core area situated at the northern edge of the Pearl River Delta.

### 3.3 Contribution of Environmental Factors

**FIGURE 5:**
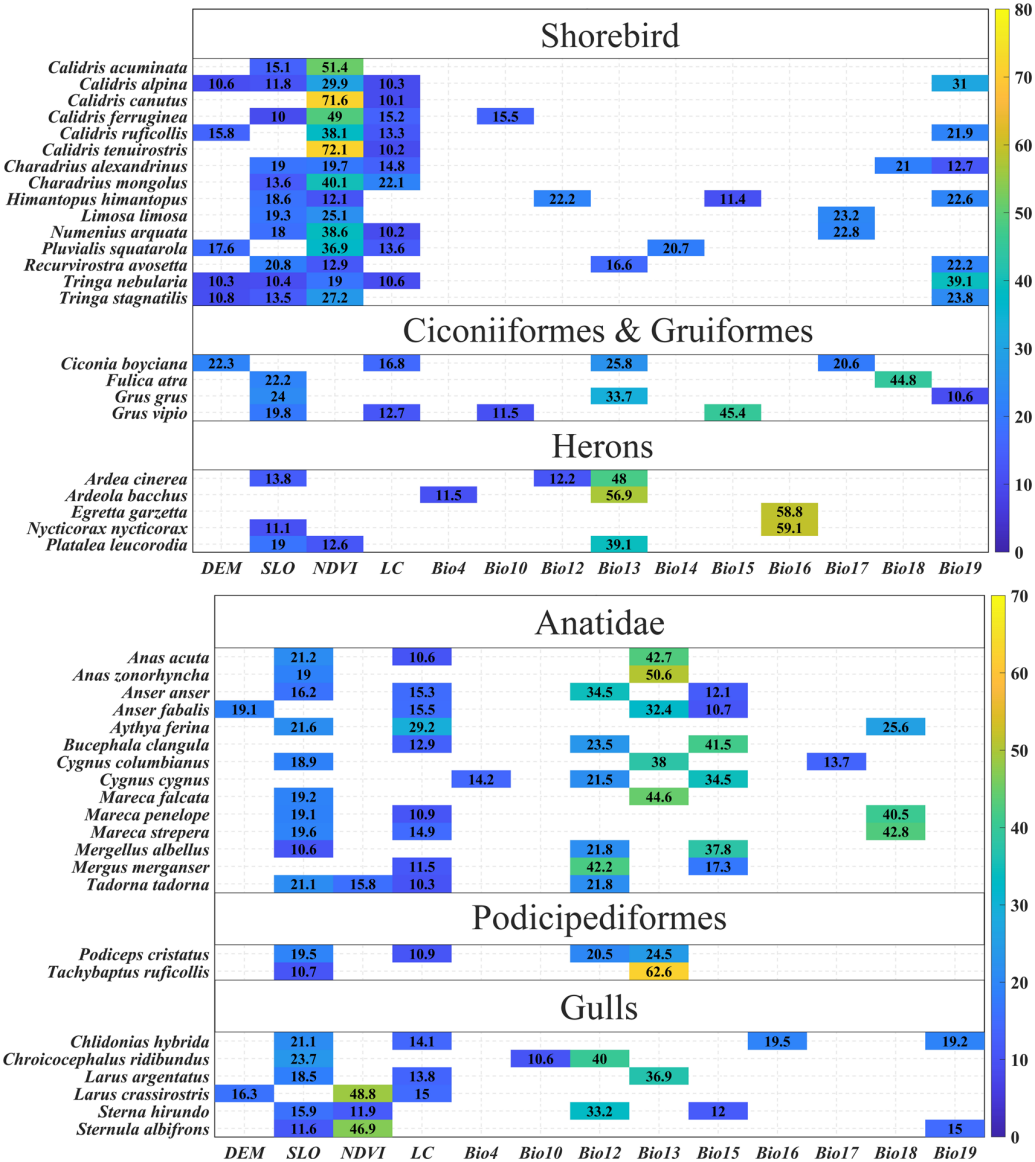
The contribution of major environmental factors to the simulation of suitable habitats for waterbirds.

The relative importance of major environmental variables (contribution rates > 10%) in predicting the habitat suitability of waterbird species is presented in Figure 5. Overall, while a broad range of environmental factors contribute to species distribution modeling, distinct differences in response patterns are evident across ecological groups though contributing less than 25% individually in most models, played important roles across the majority of species, indicating their broad but background regulatory influence on habitat suitability. In contrast, DEM showed stronger ecological group‐specific effects, with notable contributions in large‐bodied Shorebird, Ciconiiformes and Gruiformes, such as *Ciconia boyciana* and *Anser fabalis* , where DEM accounted for 22.3% and 19.1% of model contribution, respectively.

In terms of vegetation factors, NDVI played a significant role in ecological group modeling for Shorebird and Gulls, with contribution rates exceeding 70% in species such as *Calidris canutus* and *C. tenuirostris* . This result highlights the critical ecological function of sparsely vegetated habitats, such as mudflats and bare land, for these waterbirds.

Climate factors, especially precipitation‐related factors (Bio12–Bio19), were identified as dominant driving factors of ecological niche patterns, with considerable variation in sensitivity among ecological groups. Shorebird responded most strongly to Bio19, Podicipediformes were more sensitive to Bio12 and Bio13, and Anatidae exhibited a multidimensional response pattern, showing high contributions from Bio13, Bio15, and Bio18. Herons primarily responded to Bio13 and Bio16, with contribution rates exceeding 50% in some species, underscoring the importance of wet‐season water availability for maintaining suitable habitats.

Although precipitation variables were the primary climatic driving factors, some species also showed sensitivity to temperature‐related factors such as Bio4 and Bio10. These variables, though contributing less overall, may influence breeding phenology and migration timing, suggesting that climate factors shape waterbird habitat suitability not only through water availability but also via thermal regulation of life history strategies.

### 3.4 Evaluation of Key Driving Factors for Ecological Bird Groups

#### 3.4.1 Importance of Climate Factors

Using ecological groups as the unit of analysis, XGBoost was employed to systematically evaluate the feature importance of major climatic variables—originally identified in the MaxEnt models—at the group level (Table 3). Model results indicated generally strong predictive performance across groups, with high accuracy and stability of feature importance. Only the Podicipediformes exhibited relatively lower performance in both metrics, possibly reflecting greater ecological heterogeneity within the group or more complex response mechanisms to climatic driving factors.

**TABLE 3:** Mean feature importance (±SD) of climatic variables for waterbird habitat suitability by ecological group, derived using XGBoost with cross‐validation.

	Ciconiiformes & Gruiformes	Shorebird	Herons	Gulls	Podicipediformes	Anatidae
Bio4	—	—	**0.5874 ± 0.0077**	—	—	0.0736 ± 0.0004
Bio10	0.0681 ± 0.0009	0.0479 ± 0.0011	—	0.0803 ± 0.0024	—	—
Bio12	—	0.0165 ± 0.0003	0.1664 ± 0.0079	0.0150 ± 0.0033	0.3737 ± 0.4096	0.0561 ± 0.0031
Bio13	0.0141 ± 0.0008	0.0056 ± 0.0002	0.2237 ± 0.0137	0.0091 ± 0.0001	0.6263 ± 0.4096	0.0441 ± 0.0013
Bio14	—	0.0104 ± 0.0002	—	—	—	—
Bio15	0.2748 ± 0.0032	0.3074 ± 0.0029	—	0.3828 ± 0.0036	—	0.3128 ± 0.0026
Bio16	—		0.0224 ± 0.0004	0.0304 ± 0.0004	—	—
Bio17	**0.6151 ± 0.0042**	**0.5986 ± 0.0029**	—	—	—	**0.4978 ± 0.0020**
Bio18	0.0189 ± 0.0007	0.0092 ± 0.0003	—	—	—	0.0156 ± 0.0001
Bio19	0.0090 ± 0.0003	0.0044 ± 0.0001	—	**0.4788 ± 0.0028**	—	—
Overall Accuracy	0.9728	0.9817	0.9691	0.9748	0.9362	0.9696

*Note:* Values represent the mean feature importance and its standard deviation (SD) calculated across five cross‐validation folds for each ecological group. Overall accuracy corresponds to the mean prediction accuracy for each ecological group. Bold values indicate the climate variable with the highest contribution to habitat suitability for each ecological group.

Clear differences emerged among ecological groups in their response patterns to climatic variables. Ciconiiformes and Gruiformes, Shorebird, and Anatidae showed the highest feature importance for Bio17, followed by Bio15, indicating that their ecological niches are jointly shaped by the extremity and temporal variability of precipitation availability. Herons were highly sensitive to Bio4, with a feature importance score of 0.5874 ± 0.0077, underscoring the strong influence of temperature fluctuations on their physiological adaptation and breeding strategies. Gulls were primarily driven by Bio19 and Bio15, with Bio19 reaching a high importance of 0.4788 ± 0.0028, likely reflecting their strong dependence on cold‐season water resources during colony‐based breeding.

#### 3.4.2 Ecological Adaptability Assessment of Topographic and Vegetation Factors

**FIGURE 6:**
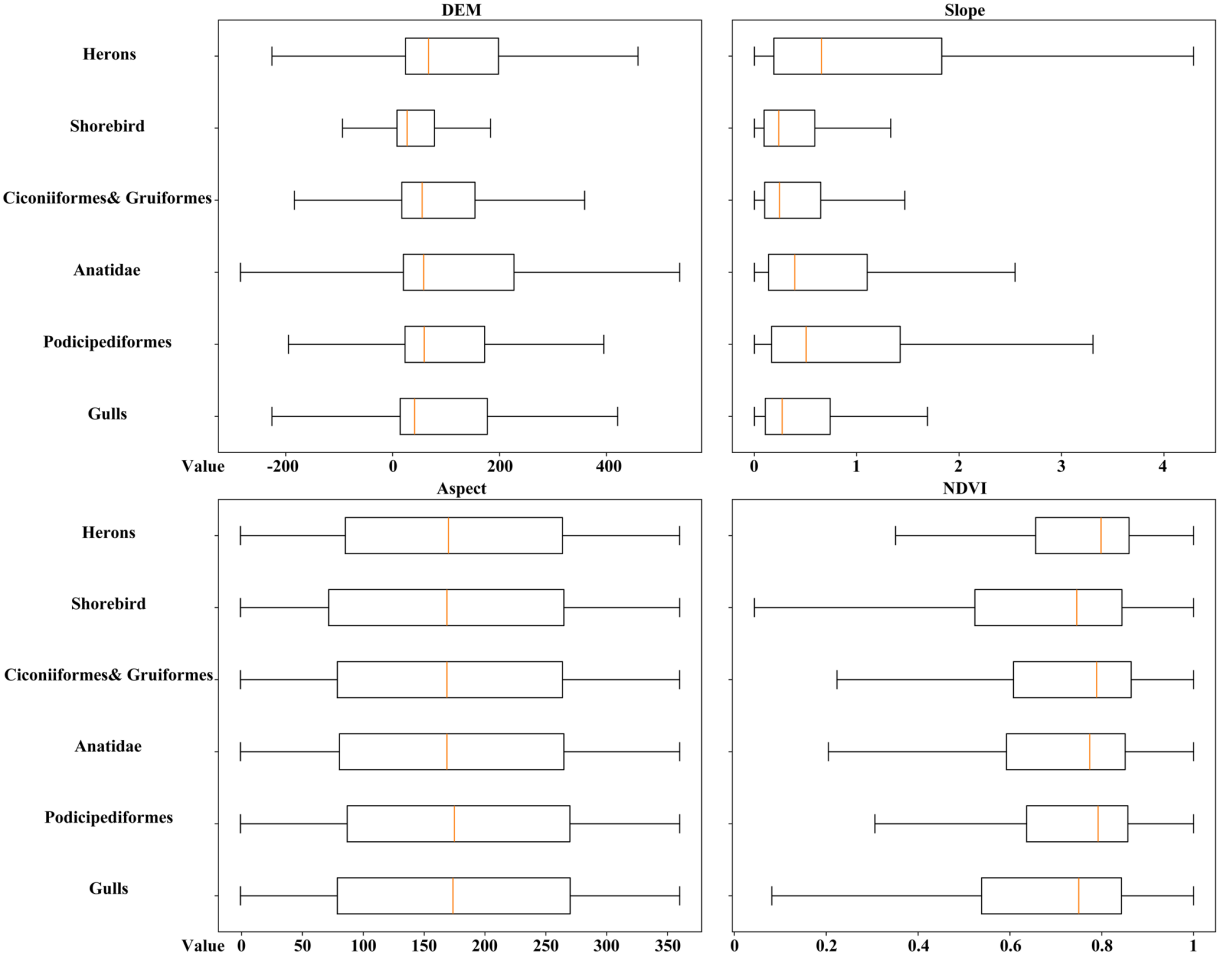
The range of natural element values in the suitable habitats for each ecological group.

We used box plots to illustrate the distribution patterns of four topographic and vegetation‐related factors within the suitable habitats of different waterbird ecological groups (Figure 6). The boxes represent the interquartile range (25%–75%), with the median and extreme values indicated. The results show distinct intergroup differences in DEM, Slope, and NDVI, while responses to Aspect were relatively consistent across groups.

In terms of DEM, Herons and Anatidae exhibited the widest range of habitats, whereas Shorebird had the narrowest distribution, and Gulls, Ciconiiformes, and Gruiformes fell within an intermediate range. For slope, all groups were primarily associated with flat areas (< 5°), with Shorebird showing the narrowest tolerance and Herons a slightly broader range. Aspect showed no clear differences among groups and was generally concentrated on east‐ to south‐facing slopes. NDVI exhibited the most pronounced variation among groups: Herons and Podicipediformes were associated with habitats characterized by high vegetation cover, Shorebird and Gulls were more frequently distributed in areas with sparse vegetation, while Ciconiiformes and Gruiformes, Anatidae occupied an intermediate position.

#### 3.4.3 Evaluation of Preferences for Land Use Factors

**FIGURE 7:**
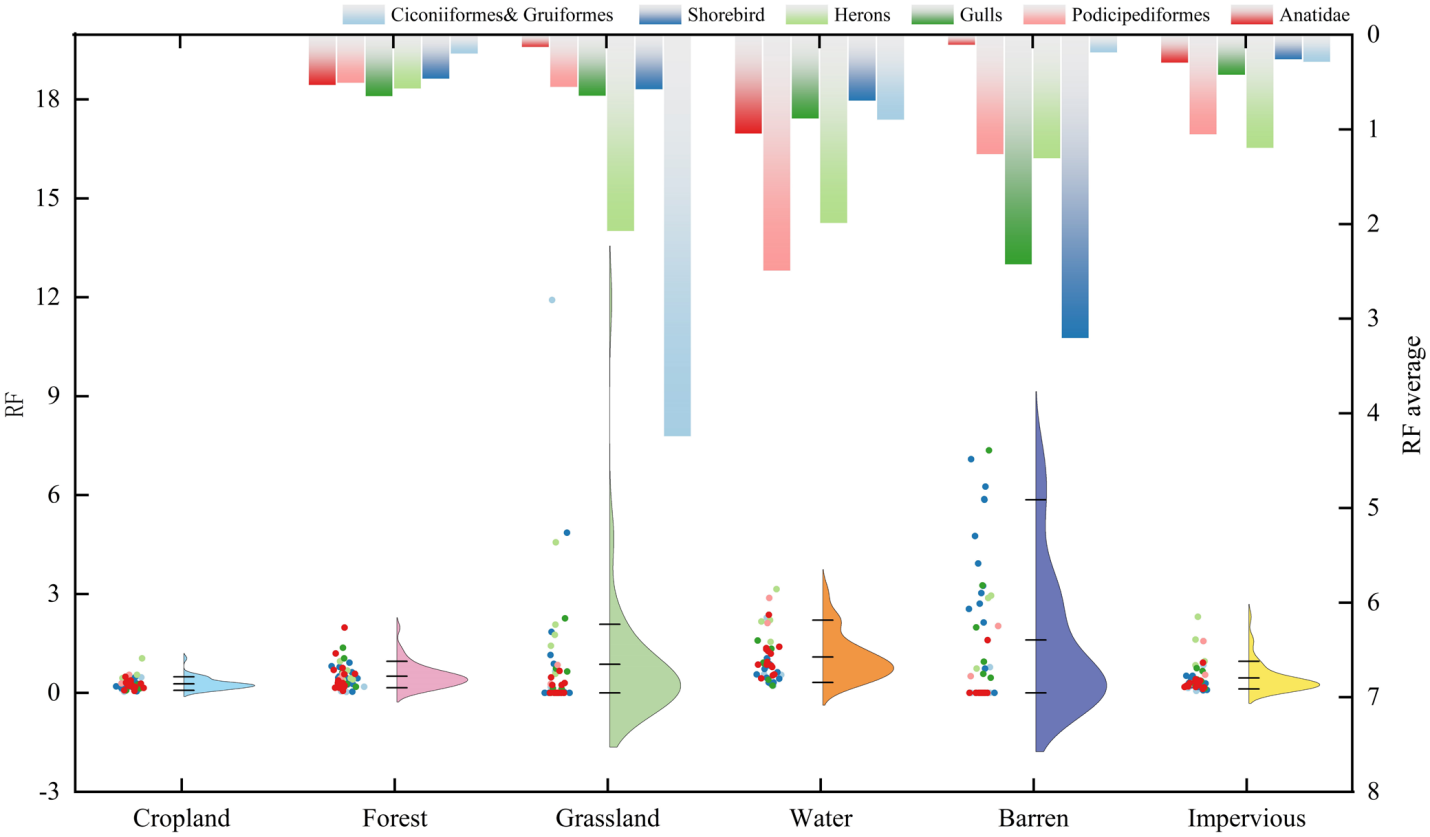
Land Cover Selection Preferences for Waterbird Habitats. Distributions and group‐level means of the Relative Preference Index (RF) across six ecological groups. RF values indicate relative land‐use associations within predicted suitable habitats, not absolute preference values.

We calculated the RF values of each ecological group for six land‐use types (Table S4) and used a composite figure to illustrate the significant differences in habitat preferences among ecological groups (Figure 7). The bar chart displays the average RF values for each group across land‐use types, while the violin plots show the distribution density of RF values, and the embedded boxplots indicate the median and interquartile ranges. Overall, barren, water, and grassland were the most preferred land types, while cropland and forest showed the lowest preference. Specifically, Ciconiiformes and Gruiformes, Herons exhibited strong preferences for grassland, reflecting their reliance on open herbaceous wetlands. Podicipediformes and Herons showed a high preference for water bodies, while Shorebird and Gulls displayed the strongest preference for barren. Notably, Herons and Podicipediformes also showed a certain degree of preference for impervious and cropland, suggesting their partial adaptability to human disturbance. While most waterbird groups tend to avoid areas with high‐intensity human activity, some groups—particularly Herons—can maintain stable distributions within semi‐urbanized landscapes.

#### 3.4.4 Response to Landscape Patterns

**FIGURE 8:**
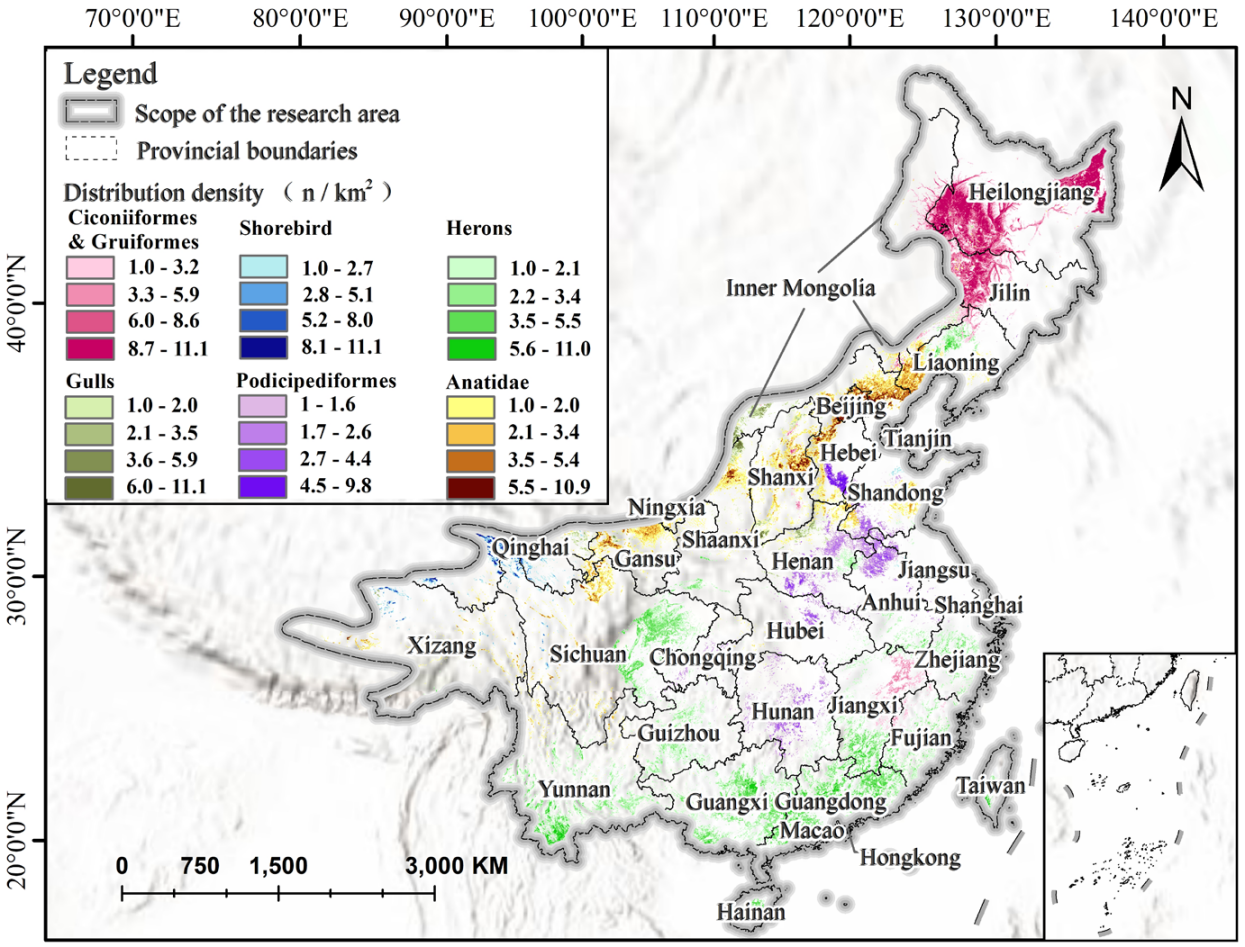
Spatial distribution of suitable habitat hotspots for different ecological groups of waterbirds.

We used the MaxEnt model prediction results for each ecological group to extract the group‐level hotspots of suitable habitats, which represent the high‐density centers of binary suitability pixels aggregated within each ecological group. Using the neighborhood analysis tool in ArcGIS, we calculated the density of these pixels and generated density grids for each group's suitable habitats (1 km^2^). In this context, “unique suitable habitats” refer to grid cells that were suitable for only one ecological group, highlighting areas of ecological specialization. This approach allowed us to quantify the spatial distribution characteristics of suitable habitats for different waterbird groups, assess the overlap between suitable habitats and human activity spaces, and identify the habitat threats and conservation needs faced by each group (Figure 8).

The results indicated that different ecological groups of waterbird exhibit distinct geographical patterns in the distribution of their suitable habitats. Suitable habitats for Ciconiiformes and Gruiformes are mainly concentrated at the borders of Heilongjiang, Jilin, and Inner Mongolia, with an additional density center located in eastern Jiangxi. Shorebird show a more scattered distribution, with some occurring in the central and southeastern margins of Qinghai Province, and small high‐density areas also found in regions such as Tianjin and Shandong. Herons show the broadest distribution, covering the southeastern coastal provinces and extending inland to Yunnan, Guizhou, Sichuan, and northwestern Liaoning. Gulls are mainly found in southern Inner Mongolia and in barren lands and riverine mudflats at the junction of Shaanxi and Shanxi provinces. Podicipediformes are concentrated in central Hunan and Henan, as well as the border regions of Hebei, Shandong, Jiangxi, and Henan. Anatidae exhibit a clear latitudinal distribution pattern, stretching from the western edge of Tibet to Bohai Bay, with sparse distribution in the west and a high‐density zone around Bohai Bay.

**FIGURE 9:**
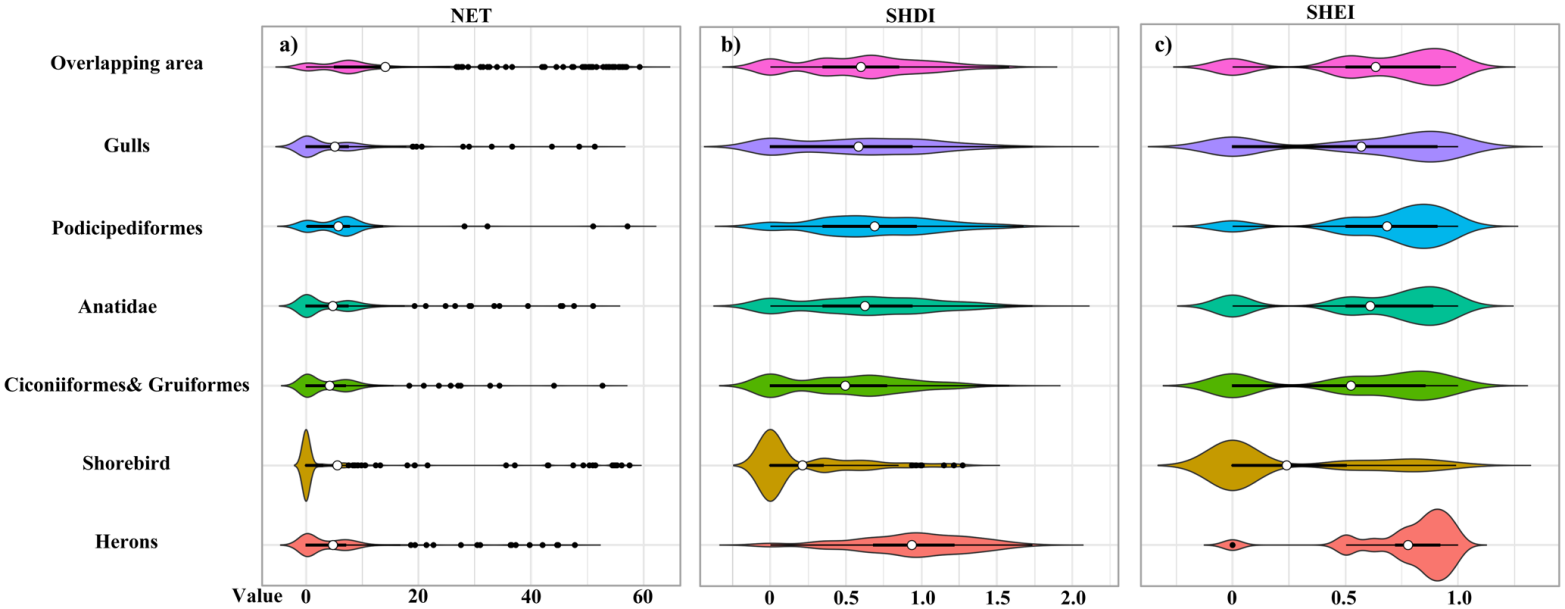
Distributions of anthropogenic and landscape indicators across ecological group hotspots. (a) Nighttime Light Index (NET) representing human disturbance intensity; (b) Shannon Diversity Index (SHDI) describing land‐use diversity; (c) Shannon Evenness Index (SHEI) reflecting the balance among land‐use types. Violin plots depict the kernel density distributions of indicator values across all 1‐km suitable habitat pixels for each ecological group.

To evaluate the exposure of suitable habitats to human disturbance and landscape heterogeneity, three indicators—Nighttime Light Index (NET), Shannon Diversity Index (SHDI), and Shannon Evenness Index (SHEI)—were analyzed for each ecological group (Figure 9).

In terms of NET (Figure 9a), values for Ciconiiformes and Gruiformes, Shorebird are concentrated in the low range, indicating their suitable habitats are mainly located in areas with low human disturbance. Podicipediformes and Herons show a wider NET range, including higher values, with suitable habitats extending into semi‐urbanized areas. Overall, overlapping regions among groups also exhibit low NET values, suggesting that shared habitats are typically located in less disturbed landscapes. For SHDI (Figure 9b), Herons show the highest values, with suitable habitats covering diverse land‐use types and complex landscape structures. Shorebird have the lowest SHDI values, with habitats dominated by single landscape types. Overlapping areas display moderately high SHDI values, reflecting greater landscape diversity that may support multi‐species coexistence. For SHEI (Figure 9c), Herons also show the highest values, indicating evenly distributed habitat types and a combined requirement for high diversity and balance. Shorebird have the lowest SHEI values, with habitats dominated by one or few types. These patterns collectively highlight contrasting ecological adaptability among groups and different tolerances to habitat heterogeneity and human disturbance.

### 3.5 Identification of Existing Protection Gaps

**FIGURE 10:**
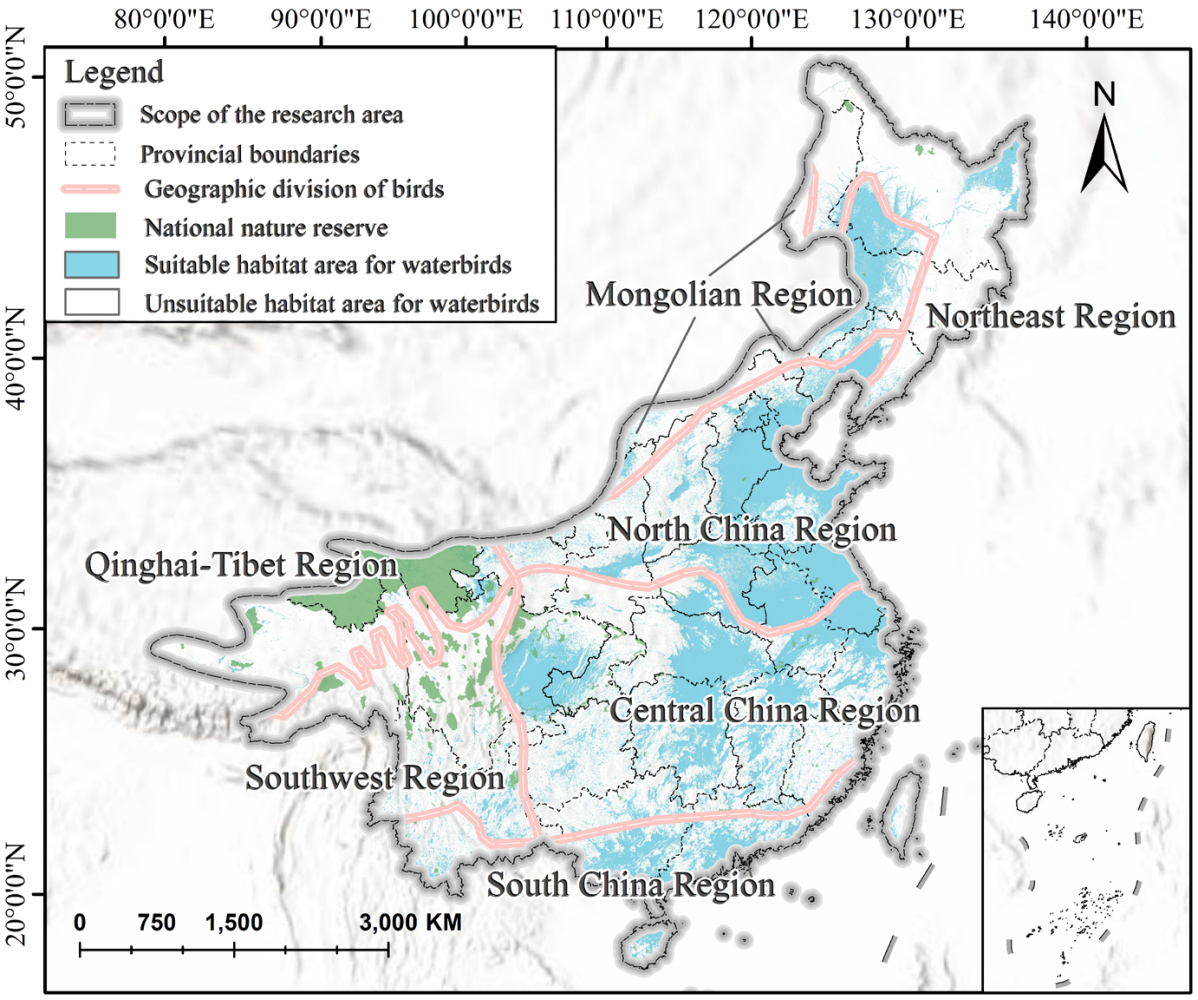
Migration routes, protected areas, and avian geographic regions within the study area.

The distribution of national nature reserves in China is uneven, with most concentrated in sparsely populated, high‐altitude areas. In contrast, protected areas in regions with high human activity and lower elevations are limited (Figure 10). Results are presented for seven bird biogeographic regions following Zheng Guangmei's geographic classification (G. Zheng 2012) to illustrate the spatial representativeness of current protection coverage. Comparisons among these regions reveal distinct differences in the spatial correspondence between suitable habitats, existing protected areas, and their overlap—particularly in regions south and east of the 800 mm precipitation line. Results are summarized in Table 4.

**TABLE 4:** Statistics of the proportion of suitable habitats and protected areas for waterbirds.

The geographic division	Suitable habitat area (10^4^Km^2^)	Nature reserve area (10^4^Km^2^)	Suitable area within reserves (10^4^Km^2^)	Percentage of suitable area within reserves (%)
North China Region	57.96	0.37	0.17	46.12%
Northeast Region	9.79	1.29	0.34	26.79%
Southwest Region	5.03	7.30	0.28	3.90%
Qinghai‐Tibet Region (Partial)	6.20	21.11	3.51	16.62%
Mongolian Region (Partial)	16.07	0.18	0.08	46.17%
Central China Region	66.93	3.50	0.63	18.05%
South China Region	24.01	0.36	0.02	6.40%

A comprehensive analysis of conservation effectiveness and spatial alignment across avifaunal regions reveals two prominent structural issues in the protection of suitable habitats for waterbirds. First, some regions contain extensive areas of suitable habitat but exhibit low protection coverage, resulting in significant conservation gaps. Second, other regions possess large areas designated as nature reserves, yet these reserves are poorly aligned with waterbird habitat distributions, indicating a spatial mismatch.

Regionally, the Central China Region hosts the largest area of suitable habitats for waterbirds (Table 4), yet only 18.05% of this area falls within nature reserves. The South China Region faces a similar challenge, with a coverage rate as low as 6.40%. These regions exemplify the pattern of “habitat‐rich but underprotected” and urgently require an expansion in the number and density of protected areas to mitigate risks associated with habitat loss and anthropogenic disturbances.

In contrast, the Qinghai–Tibet and Southwest regions, despite possessing large areas of nature reserves (21.11 and 7.30 × 10^4^ km^2^), show limited overlap with suitable habitats for waterbirds, with coverage rates of only 16.62% and 3.90%. This indicates a pronounced spatial mismatch, suggesting that the current conservation system in these regions may be more focused on high‐altitude ecosystems and large mammals, with insufficient consideration of wetland birds' habitat needs.

In other regions, conservation effectiveness is relatively higher. The North China and Northeast regions demonstrate good spatial alignment between protected areas and suitable habitats, with coverage rates of 46.12% and 26.79%, respectively. Particularly in North China, a high coverage ratio has been achieved despite the region's substantial habitat extent, serving as a valuable example of effective spatial planning. Additionally, in the Mongolian Region (partial), although the total protected area is limited, 46.17% of the suitable habitats are covered, indicating well‐targeted conservation planning and notable protection outcomes under constrained conditions.

## 4 Discussion

Landscape composition and environmental variability shape the complexity and availability of ecological niches, thereby structuring biodiversity patterns across spatial scales. Understanding how different environmental gradients influence waterbird habitat suitability provides critical insights into their ecological adaptation and conservation priorities.

### 4.1 Model Performance and Spatial Patterns of Suitable Habitats

Model robustness forms the foundation for reliable species habitat predictions and the development of effective conservation strategies (Feng, Park, Walker, et al. 2019). In this study, all MaxEnt models for the 46 waterbird species—except for *Ichthyaetus relictus* —demonstrated high predictive performance (AUC > 0.90) and strong stability (CV < 15%), indicating that the selected environmental variables effectively captured the key ecological gradients influencing waterbird habitat selection. The notably lower performance for *I. relictus* likely reflects a combination of factors, including a limited effective sample size after quality control and spatial thinning, spatial clustering of observations around a few reporting hotspots, and a temporal mismatch between single‐year citizen‐science occurrences and the multi‐year climatic normals used as predictors, which can obscure responses of highly mobile, colony‐forming larids to short‐term hydrological states (Boria et al. 2014; Wisz et al. 2008; Phillips et al. 2009; Kramer‐Schadt et al. 2013). Consistent with our a priori reliability criteria, this species was excluded from subsequent analyses.

Although MaxEnt is suitable for modeling with limited species data, its performance may still be compromised when applied across large spatial extents with sparse occurrence records (Phillips et al. 2006), and when the sample size is extremely small, the model may yield spuriously high accuracy due to overfitting or insufficient environmental representation, and therefore its reliability should be carefully evaluated in specific contexts (Wisz et al. 2008). After mapping and comparing the spatial distribution of occurrence records for other low‐sample species (e.g., *Calidris tenuirostris, C. canutus*) with those of *I. relictus* , we found that *C. tenuirostris* and *C. canutus* achieved relatively higher model accuracy, which may be partly related to their ecological traits. Compared with *I. relictus* , *C. tenuirostris* and *C. canutus* have more dispersed distributions across multiple coastal stopover sites and are exposed to a broader range of environmental conditions. In contrast, *I. relictus* shows strong site fidelity and colony‐based breeding behavior, with occurrences concentrated in a few habitats and temporally unstable, which limits the environmental variability available for model calibration and may therefore result in lower predictive accuracy.

The models successfully revealed critical spatial patterns of habitat suitability for waterbirds. Distinct hotspots were identified along coastal areas such as Tianjin, Shanghai, and the northern margin of the Pearl River Delta. These coastal zones, serving as vital nodes along the EAAF, provide consistent ecological resources—such as tidal wetlands and abundant benthic communities—that offer stable and continuous habitats for waterbird populations (Takekawa et al. 2023; Lei et al. 2019). In contrast, inland hotspots in provinces such as Hubei, Hunan, Jiangxi, and Anhui were predominantly distributed along major river systems, highlighting the critical role of hydrological dynamics in supporting wetland habitats in inland regions. Seasonal flood regimes and periodic water‐level fluctuations in these wetlands increase habitat complexity, offering diverse and dynamic ecological niches for various waterbird species and promoting ecosystem stability and adaptive capacity (Qiu et al. 2024; Studds et al. 2012).

### 4.2 Ecological Mechanisms Shaping Waterbirds Niches

Topographic factors significantly influence the distribution and utilization of ecological niches. The widespread presence of waterbird habitats in low‐slope areas indicates that gentle slopes play a key ecological role in maintaining stable hydrological conditions. Such hydrological stability enhances the predictability of flooding and recession cycles, in turn modulating hydroperiod and shoreline geometry. A stable water‐level–shoreline configuration sustains persistent aquatic vegetation and dense benthic invertebrate assemblages, thereby increasing foraging opportunities and energetic returns for waterbirds (Deumlich et al. 2021). In contrast, slope aspect exerted a limited influence on habitat distribution, suggesting that waterbirds exhibit behavioral flexibility and ecological adaptability in habitat utilization (Henriques et al. 2022).

DEM and NDVI showed particularly high contributions to niche modeling for shorebird, reflecting their specialized ecological adaptations. Shorebird preferentially occupy open, low‐elevation areas with sparse vegetation, which provide unobstructed sightlines and accessible soft substrates, thereby facilitating foraging and predator avoidance (Henriques et al. 2022). For species such as *Calidris canutus* and *Calidris tenuirostris* , elevated primary productivity in adjacent vegetated zones (indexed by higher NDVI) can subsidize benthic prey production on nearby flats, revealing a trophic linkage between vegetation productivity and benthic prey availability (Qiu et al. 2024). Taken together, DEM controls where shallow water and drawdown margins form, while NDVI reflects how vegetation structure mediates prey concentration and concealment—two pathways that connect landscape setting to realized foraging conditions.

Climatic factors further shape the ecological niches of waterbirds by modulating precipitation dynamics. For instance, Bio13 is particularly important for species like *Ardeola bacchus* and *Anas zonorhyncha* , which are highly dependent on shallow wetlands—habitats that are sensitive to seasonal extremes in precipitation (Wypych and Ustrnul 2024). Meanwhile, Bio16 supports the long‐term stability of wetland ecosystems, directly determining habitat suitability for species such as *Egretta garzetta* and *Nycticorax nycticorax* . Although both are precipitation‐related variables, they represent distinct ecological processes: Bio13 influences within‐season water availability that governs nest initiation and brood‐rearing water, whereas Bio16 underpins multi‐season wetland resilience and food‐web stability (Studds et al. 2012). In shorebird systems, Bio17 constrains the frequency and duration of mudflat exposure, thereby defining the temporal window for effective foraging.

### 4.3 Group‐Specific Ecological Responses and Adaptations

Building upon the species‐level suitability results from MaxEnt, this study applied XGBoost analysis to further elucidate the ecological adaptation strategies of different waterbird groups, thereby extending species‐level predictions to a broader ecological context. For instance, Ciconiiformes and Gruiformes, shorebird, and Anatidae exhibited strong sensitivity to Bio17, followed by Bio15. Functionally, this pattern indicates that seasonal rainfall variability controls the creation and persistence of shallow foraging zones and seed/invertebrate pulses, thereby modulating foraging window length and resource reliability, an adaptive response to resource uncertainty (Goetz et al. 2014). In contrast, herons showed pronounced sensitivity to Bio4, highlighting the ecological relevance of thermal variation in shaping their reproductive strategies (Kelly and Condeso 2014). Fluctuations in temperature seasonality influence breeding phenology, nest initiation, and chick thermoregulation, and together with precipitation controls, affect colony stability and nesting success.

Independently of climatic effects and model‐specific responses, the landuse types further reflect anthropogenic trade‐offs in ecological adaptation. Shorebird and gulls prefer barren habitats, where unobstructed sightlines enhance search rate and predator detection and where exposed sediments facilitate pecking, while winter freshwater and estuarine inputs (linked to Bio19) support fish availability for gulls or terns in non‐breeding and early breeding periods (Santos et al. 2023). In contrast, Ciconiiformes and Gruiformes, Herons exhibit a preference for grasslands, which tend to provide richer insect resources, more stable vegetation structures, and reduced human disturbance levels (Whittingham and Devereux 2008). These preferences map onto the mechanisms above: vegetated shallows provide structural cover and prey refugia for wading and diving foragers, whereas open substrates maximize visibility and access to infaunal prey. These ecological distinctions underscore the critical role of habitat complexity and landscape heterogeneity in sustaining ecological diversity.

### 4.4 Landscape Structure, Human Disturbance, and Habitat Quality

Analyses of SHDI and SHEI indices revealed key characteristics of habitat quality and ecological resilience. The low landscape diversity index of shorebird habitats indicates a high degree of ecological specialization, rendering them more vulnerable to the negative effects of habitat simplification (Xia et al. 2017; Wang et al. 2014). In contrast, the high diversity and evenness observed in heron habitats underscore their broader ecological adaptability and stronger resilience.

The clustering of suitable habitats in areas with low NET indicates the general sensitivity of waterbirds to human disturbance, consistent with previous studies (de Jong 2016). However, certain herons and podicipediform species exhibited a degree of ecological tolerance and even preference for habitats on the urban periphery. This suggests that some species may possess ecological plasticity, allowing them to exploit human‐modified environments. These peri‐urban wetlands are often artificially managed, providing stable water levels and predictable food resources, which support the persistence of more adaptable species (Dominoni 2015).

### 4.5 Conservation Gaps and Management Priorities

Despite China's extensive network of nature reserves, this study reveals pronounced spatial imbalances and structural inefficiencies in the protection of suitable habitats for waterbirds across different avifaunal regions. In areas such as Central and South China, vast stretches of suitable habitats remain largely unprotected, exposing significant conservation gaps. In contrast, although the Qinghai–Tibet Plateau and Southwest China have designated large reserve areas, these are poorly aligned with the actual ecological needs of waterbirds, leading to clear spatial mismatches.

These mismatches stem from a combination of ecological and historical factors. On the one hand, regions rich in habitat resources often coincide with areas of intensive human activity, where urban expansion and agricultural intensification have caused severe habitat fragmentation and undermined the effectiveness of existing reserve networks. On the other hand, the early establishment of China's reserve system was primarily oriented toward forest ecosystems and large terrestrial mammals, resulting in the underrepresentation of wetland‐associated bird species and their seasonal habitats in conservation planning. Furthermore, inland river–lake wetlands and coastal intertidal zones have long lacked coherent ecological corridor designs and integrated restoration strategies, further exacerbating the discontinuity of habitats essential to migratory species. In light of these spatial and structural mismatches, there is an urgent need to establish a dual‐pathway conservation framework that focuses on optimizing spatial layout and restoring ecological functionality.

The optimization of spatial layout should prioritize two key regional categories: one comprises areas rich in habitat resources but with sparse protection coverage, and the other includes regions with substantial reserve areas that are poorly aligned with the ecological needs of waterbirds. The former requires expanded protection coverage, increased reserve density, and the implementation of flexible spatial zoning mechanisms to enhance coverage of critical habitats. The latter calls for the reintegration of habitat components and the recalibration of reserve functions to better serve waterbirds' habitat use and migratory connectivity. Within this process, the spatial demands of long‐distance migratory species offer important guidance. Species such as *Anser indicus* and *Ciconia boyciana* are highly dependent on the continuity and phenological synchrony of transregional stopover site networks. In recent years, these species have faced increasing challenges such as stopover site fragmentation, habitat chain disjunction, and temporal mismatches between migration windows and protected areas—factors that have significantly weakened the functional continuity of their migratory routes (Shi et al. 2024; Liu et al. 2008). Existing studies have demonstrated that such mismatches not only cause local population declines but also lead to systematic advancement in departure timing, reflecting deeper ecological impacts of habitat discontinuity (Aarif et al. 2021). Therefore, the spatial planning and construction of ecological corridors and stopover sites should be systematically incorporated into national spatial planning frameworks and precisely aligned with the phenological characteristics of migratory species in order to enhance the accessibility and adaptability of migration networks (Takekawa et al. 2023).

At the same time, reinforcing ecological functionality constitutes the second core pathway for achieving ecosystem restoration and targeted species protection. Specialized groups such as shorebird are highly dependent on open microhabitats such as barren lands and intertidal flats, which are rapidly disappearing due to coastal development and wetland degradation (Santos et al. 2023). Recent studies have shown that declines in shorebird populations and advances in migration departure times signal disruptions in seasonal habitat suitability and the functional integrity of migratory networks (Aarif et al. 2021). Therefore, targeted restoration and adaptive management that ensure both seasonal suitability and spatial continuity of wetlands should be implemented in the identified priority regions. In addition, wetlands—as critical ecological systems—not only provide essential habitats for waterbirds but also play foundational roles in hydrological regulation, biodiversity maintenance, and regional climate resilience (Ma et al. 2024). In ecologically stressed regions with high‐intensity human activity, such as North China, restoring and reconstructing wetland functionality is essential not only for enhancing the habitat capacity for waterbirds but also for improving ecosystem service delivery and environmental stability at the regional scale (Ouyang et al. 2019). Looking ahead, sustaining the effectiveness of these dual conservation pathways will require continuous adaptation to emerging climatic and environmental dynamics. Increasing temperature seasonality and altered precipitation regimes are expected to reshape wetland extent and timing, potentially disrupting habitat availability for migratory species. Integrating climate projections and hydrological scenarios into regional conservation planning, while maintaining flexibility in corridor design and restoration priorities, will be essential to ensure the long‐term resilience of waterbird habitats and their supporting ecosystems.

## 5 Conclusion

The study used MaxEnt and XGBoost to assess waterbird habitat suitability in the region east and south of the 800‐mm isohyet. Different groups responded differently to topographic, climatic, vegetative, and land‐use factors, with DEM, NDVI, precipitation, and temperature seasonality as the main driving factors. Generalist species showed greater tolerance to human disturbance and landscape change, whereas specialists (e.g., *Ichthyaetus relictus* ) were more sensitive to habitat conditions. The conservation assessment revealed a clear mismatch between high‐suitability habitats and existing protected areas, especially in South and Southwest China. Priority actions should focus on expanding protected areas, restoring degraded wetlands, and improving landscape connectivity to increase conservation effectiveness.

This study provides a robust assessment of current habitat suitability and conservation gaps, yet species distributions remain dynamic under ongoing climate change. Although the framework applied here is effective for identifying present‐day patterns, it has limited capacity to project future shifts, and the spatial resolution suitable for regional planning may not capture fine‐scale refugia or subtle habitat alterations. The temporal design also introduces constraints, as reliance on 2022 occurrence records together with climatological or multi‐year environmental layers reduces the ability to detect annual anomalies. In particular, extreme climatic events after 2020 may not be fully represented, and using only one year of bird data restricts insights into interannual dynamics. To strengthen predictive value and long‐term relevance, future research should incorporate scenario‐based simulations that integrate projected climate and land‐use change, expand occurrence data to multi‐year series, and make use of higher‐temporal‐resolution environmental variables. Approaches that combine temporal cross‐validation and sensitivity analyses will improve understanding of consistency across years, help anticipate potential range shifts and refugia, and provide a stronger scientific basis for proactive conservation planning in regions east and south of the 800‐mm precipitation isohyet.

## Author Contributions

**Jiaxu Fan:** conceptualization (lead), formal analysis (lead), methodology (lead), writing – original draft (lead), writing – review and editing (equal). **Peng Du:** conceptualization (equal), formal analysis (equal), methodology (equal), validation (equal), visualization (lead). **Yi Lian:** conceptualization (equal), funding acquisition (supporting), supervision (equal), validation (lead), writing – review and editing (equal). **Lei Cui:** funding acquisition (supporting), resources (supporting). **Haixiao Li:** data curation (equal), resources (supporting). **Long He:** data curation (equal), visualization (supporting). **Yuanyuan Tan:** data curation (equal), visualization (equal). **Xunqiang Mo:** formal analysis (equal), funding acquisition (lead), methodology (equal), project administration (equal), supervision (equal), writing – review and editing (equal). **Zhengwang Zhang:** project administration (lead), supervision (lead), writing – review and editing (lead).

## Funding

This research was funded by the National Natural Science Foundation of China (grant no. 42501422), Tianjin Normal University (grant no. 52XB1618), Tianjin Municipal Education Commission (grant no. 2023SK030), and Natural Science Foundation of Xiamen Municipality (grant no. 3502Z202473059).

## Conflicts of Interest

The authors declare no conflicts of interest.

## Supporting information


**Appendix S1:** ece372730‐sup‐0001‐AppendixS1.docx.

**Data S1:** ece372730‐sup‐0002‐DataS1.rar.

## Data Availability

The environmental variables used in this study were obtained from publicly accessible databases, with all data sources properly cited in the Methods section. Species distribution data, MaxEnt modeling results (representative habitat suitability maps), and the analysis code have been uploaded to the ScholarOne submission system for peer review purposes. Due to the large file size (approximately 200 GB), the full MaxEnt outputs for all 46 species have not been publicly archived but are available from the authors upon reasonable request. All data and scripts required to reproduce the main analyses and figures are provided as Supporting Information.
