# Supplementary material for: Flyway‐Scale Assessment of Habitat Suitability and Key Environmental Drivers for Waterbirds in Southern China
Source: Ecol Evol. 2025 Dec 23;15(12):e72730. doi: 10.1002/ece3.72730 (PMC12723443; doi:10.1002/ece3.72730)
Supplement: Supplementary file 1 — Appendix S1: ece372730‐sup‐0001‐AppendixS1.docx. [file ECE3-15-e72730-s002.docx]

**Appendix：**

**Table S1. Bird Point Records and Model Prediction Accuracy (AUC, dimensionless)**

| **ID** | **Species** | **Number of points** | **Model accuracy** | **Habitat suitability threshold** |
| --- | --- | --- | --- | --- |
| **Waterfowl-Anatidae** | | | | |
| 1 | *Anas acuta* | 158 | 0.955 | 0.374558 |
| 2 | *Anas zonorhyncha* | 698 | 0.950 | 0.355985 |
| 3 | *Anser anser* | 121 | 0.954 | 0.359058 |
| 4 | *Anser fabalis* | 110 | 0.978 | 0.384328 |
| 5 | *Aythya ferina* | 198 | 0.965 | 0.361561 |
| 6 | *Bucephala clangula* | 75 | 0.939 | 0.356319 |
| 7 | *Cygnus columbianus* | 113 | 0.970 | 0.408582 |
| 8 | *Cygnus cygnus* | 64 | 0.943 | 0.322201 |
| 9 | *Mareca faLCata* | 224 | 0.970 | 0.369396 |
| 10 | *Mareca penelope* | 185 | 0.971 | 0.372306 |
| 11 | *Mareca strepera* | 265 | 0.957 | 0.353025 |
| 12 | *Mergellus albellus* | 100 | 0.968 | 0.339988 |
| 13 | *Mergus merganser* | 231 | 0.963 | 0.337711 |
| 14 | *Tadorna tadorna* | 98 | 0.963 | 0.376796 |
| **Waterfowl-Podicipediformes** | | | | |
| 15 | *Podiceps cristatus* | 459 | 0.950 | 0.344948 |
| 16 | *Tachybaptus ruficollis* | 1200 | 0.928 | 0.339603 |
| **Waterfowl-Gulls** | | | | |
| 17 | *Chlidonias hybrida* | 194 | 0.960 | 0.35984 |
| 18 | *Chroicocephalus ridibundus* | 298 | 0.963 | 0.342737 |
| 19 | *Ichthyaetus relictus* | 9 | 0.872 | 0.405373 |
| 20 | *Larus vegae* | 251 | 0.966 | 0.361453 |
| 21 | *Larus crassirostris* | 58 | 0.958 | 0.373225 |
| 22 | *Sterna hirundo* | 173 | 0.954 | 0.346735 |
| 23 | *Sternula albifrons* | 51 | 0.915 | 0.348336 |
| **Waders-Shorebird** | | | | |
| 24 | *Calidris acuminata* | 44 | 0.859 | 0.51219 |
| 25 | *Calidris alpina* | 90 | 0.970 | 0.388871 |
| 26 | *Calidris canutus* | 19 | 0.985 | 0.396445 |
| 27 | *Calidris ferruginea* | 42 | 0.928 | 0.386105 |
| 28 | *Calidris ruficollis* | 69 | 0.910 | 0.385358 |
| 29 | *Calidris tenuirostris* | 23 | 0.889 | 0.391452 |
| 30 | *Charadrius alexandrinus* | 173 | 0.963 | 0.380288 |
| 31 | *Charadrius mongolus* | 75 | 0.923 | 0.365645 |
| 32 | *Himantopus himantopus* | 284 | 0.956 | 0.359331 |
| 33 | *Limosa limosa* | 92 | 0.973 | 0.368606 |
| 34 | *Numenius arquata* | 52 | 0.950 | 0.378417 |
| 35 | *Pluvialis squatarola* | 50 | 0.965 | 0.381622 |
| 36 | *Recurvirostra avosetta* | 167 | 0.964 | 0.372635 |
| 37 | *Tringa nebularia* | 260 | 0.965 | 0.382157 |
| 38 | *Tringa stagnatilis* | 102 | 0.980 | 0.355683 |
| **Waders-Ciconiiformes and Gruiformes** | | | | |
| 39 | *Ciconia boyciana* | 64 | 0.957 | 0.385488 |
| 40 | *Fulica atra* | 618 | 0.948 | 0.330264 |
| 41 | *Grus grus* | 61 | 0.979 | 0.405469 |
| 42 | *Grus vipio* | 34 | 0.974 | 0.375592 |
| **Waders-Herons** | | | | |
| 43 | *Ardea cinerea* | 869 | 0.934 | 0.339819 |
| 44 | *Ardeola bacchus* | 729 | 0.945 | 0.347994 |
| 45 | *Egretta garzetta* | 1442 | 0.921 | 0.33879 |
| 46 | *Nycticorax nycticorax* | 831 | 0.945 | 0.32843 |
| 47 | *Platalea leucorodia* | 165 | 0.964 | 0.388496 |

**Table S2. Datasets, name remarks, descriptions, temporal coverage, and DOI/URL used in this study**

| Dataset | **Name Remarks** | **Description** | **Temporal coverage** | **DOI/URL** |
| --- | --- | --- | --- | --- |
| World Climate v2.1 Bioclim | Bio1-Bio19 | 19 bioclimatic variables describing temperature and precipitation patterns. | 1970–2000 | <https://doi.org/10.1002/joc.5086> |
| Copernicus DEM GLO-30 | DEM | Digital Elevation Model. | 2010–2015 | <https://doi.org/10.5270/ESA-c5d3d65> |
| Copernicus DEM GLO-30 (derived) | SLO | Topographic slope represents terrain steepness affecting hydrological and vegetation patterns. | 2010–2015 | Derived from DEM |
| Copernicus DEM GLO-30 (derived) | ASP | Topographic aspect used to represent topographic exposure and microclimatic variation. | 2010–2015 | Derived from DEM |
| China 30-m Annual-Max NDVI | NDVI | Vegetation greenness index, representing vegetation cover/condition. | 2022 | https://doi.org/10.12199/nesdc.ecodb.rs.2021.012 |
| The 30 m annual land cover dataset | LC | Categorical land-use. | 2022 | https://doi.org/10.5281/zenodo.4417809 |
| China statistical yearbook | POP | Population density. | 2023 | https://www.stats.gov.cn/sj/ndsj/2023/indexch.htm |
| China Nighttime Lights Annual Dataset | NET | Night-time light intensity as a proxy for human activity intensity. | 2022 | https://doi.org/10.12078/2022090902 |
| National Conservation Areas | N/A | Official nature reserve boundaries used for conservation-gap analysis. | Up to 2018 | https://www.resdc.cn/data.aspx?DATAID=272 |

**Table S3. Final Retained Variables for Modeling Each Species**

| **Species** | **Final Variables Used in MaxEnt Modeling** |
| --- | --- |
| ***Waterfowl-Anatidae*** | |
| *Anas acuta* | Bio3, Bio7, Bio8, Bio9, Bio13, Bio15, Bio19, DEM, SLO, ASP, NDVI, POP, LC |
| *Anas zonorhyncha* | Bio3, Bio7, Bio9, Bio10, Bio13, Bio15, Bio19, DEM, SLO, ASP, NDVI, POP, LC |
| *Anser anser* | Bio2, Bio4, Bio6, Bio10, Bio12, Bio15, Bio18, DEM, SLO, ASP, NDVI, POP, LC |
| *Anser fabalis* | Bio8, Bio9, Bio11, Bio13, Bio15, Bio16, DEM, SLO, ASP, NDVI, POP, LC |
| *Aythya ferina* | Bio2, Bio3, Bio4, Bio8, Bio9, Bio15, Bio18, Bio19, DEM, SLO, ASP, NDVI, POP, LC |
| *Bucephala clangula* | Bio3, Bio7, Bio8, Bio9, Bio12, Bio14, Bio15, DEM, SLO, ASP, NDVI, POP, LC |
| *Cygnus columbianus* | Bio4, Bio13, Bio15, Bio17, DEM, SLO, ASP, NDVI, POP, LC |
| *Cygnus cygnus* | Bio2, Bio4, Bio8, Bio9, Bio12, Bio15, DEM, SLO, ASP, NDVI, POP, LC |
| *Mareca faLCata* | Bio1, Bio3, Bio7, Bio8, Bio11, Bio13, Bio15, Bio19, DEM, SLO, ASP, NDVI, POP, LC |
| *Mareca penelope* | Bio3, Bio7, Bio10, Bio11, Bio15, Bio18, Bio19, DEM, SLO, ASP, NDVI, POP, LC |
| *Mareca strepera* | Bio2, Bio3, Bio4, Bio6, Bio10, Bio15, Bio18, Bio19, DEM, SLO, ASP, NDVI, POP, LC |
| *Mergellus albellus* | Bio3, Bio7, Bio8, Bio9, Bio12, Bio15, Bio18, Bio19, DEM, SLO, ASP, NDVI, POP, LC |
| *Mergus merganser* | Bio2, Bio3, Bio7, Bio8, Bio9, Bio12, Bio15, Bio18, DEM, SLO, ASP, NDVI, POP, LC |
| *Tadorna tadorna* | Bio2, Bio3, Bio6, Bio10, Bio12, Bio15, Bio18, Bio19, DEM, SLO, ASP, NDVI, POP, LC |
| **Waterfowl-Podicipediformes** | |
| *Podiceps cristatus* | Bio2, Bio3, Bio4, Bio9, Bio10, Bio12, Bio13, Bio15, DEM, SLO, ASP, NDVI, LC |
| *Tachybaptus ruficollis* | Bio1, Bio3, Bio7, Bio8, Bio13, Bio15, Bio19, DEM, SLO, ASP, NDVI, POP, LC |
| **Waterfowl-Gulls** | |
| *Chlidonias hybrida* | Bio3, Bio4, Bio6, Bio10, Bio15, Bio16, Bio19, DEM, SLO, ASP, NDVI, POP, LC |
| *Chroicocephalus ridibundus* | Bio2, Bio3, Bio4, Bio9, Bio10, Bio12, Bio15, Bio19, DEM, SLO, ASP, NDVI, POP, LC |
| *Ichthyaetus relictus* | Bio15, DEM, SLO, NDVI, POP, LC |
| *Larus vegae* | Bio3, Bio5, Bio7, Bio9, Bio12, Bio13, Bio15, DEM, SLO, ASP, NDVI, POP, LC |
| *Larus crassirostris* | Bio2, Bio3, Bio6, Bio14, DEM, SLO, ASP, NDVI, POP, LC |
| *Sterna hirundo* | Bio2, Bio3, Bio4, Bio8, Bio9, Bio12, Bio13, Bio15, DEM, SLO, ASP, NDVI, POP, LC |
| *Sternula albifrons* | Bio2, Bio6, Bio10, Bio15, Bio16, Bio19, DEM, SLO, ASP, NDVI, POP, LC |
| **Waders-Shorebird** | |
| *Calidris acuminata* | Bio2, Bio3, Bio9, Bio16, Bio19, DEM, SLO, ASP, NDVI, POP, LC |
| *Calidris alpina* | Bio3, Bio9, Bio10, Bio15, Bio19, DEM, SLO, ASP, NDVI, POP, LC |
| *Calidris canutus* | Bio9, Bio15, DEM, SLO, ASP, NDVI, POP, LC |
| *Calidris ferruginea* | Bio8, Bio9, Bio15, DEM, SLO, ASP, NDVI, POP, LC |
| *Calidris ruficollis* | Bio9, Bio10, Bio19, DEM, SLO, ASP, NDVI, POP, LC |
| *Calidris tenuirostris* | Bio2, Bio11, Bio14, Bio15, DEM, SLO, ASP, NDVI, POP, LC |
| *Charadrius alexandrinus* | Bio2, Bio3, Bio6, Bio15, Bio18, Bio19, DEM, SLO, ASP, NDVI, POP, LC |
| *Charadrius mongolus* | Bio4, Bio8, Bio9, Bio15, Bio17, DEM, SLO, ASP, NDVI, LC |
| *Himantopus himantopus* | Bio3, Bio4, Bio6, Bio10, Bio15, Bio19, DEM, SLO, ASP, NDVI, POP, LC |
| *Limosa limosa* | Bio2, Bio3, Bio6, Bio10, Bio15, Bio17, Bio18, DEM, SLO, ASP, NDVI, POP, LC |
| *Numenius arquata* | Bio2, Bio8, Bio9, Bio15, Bio17, DEM, SLO, ASP, NDVI, POP, LC |
| *Pluvialis squatarola* | Bio2, Bio3, Bio6, Bio12, Bio14, Bio19, DEM, SLO, ASP, NDVI, POP, LC |
| *Recurvirostra avosetta* | Bio2, Bio3, Bio9, Bio10, Bio13, Bio15, Bio19, DEM, SLO, ASP, NDVI, POP, LC |
| *Tringa nebularia* | Bio3, Bio4, Bio9, Bio10, Bio15, Bio19, DEM, SLO, ASP, NDVI, POP, LC |
| *Tringa stagnatilis* | Bio1, Bio3, Bio6, Bio7, Bio15, Bio19, DEM, SLO, ASP, NDVI, POP, LC |
| **Waders-Ciconiiformes and Gruiformes** | |
| *Ciconia boyciana* | Bio6, Bio8, Bio13, Bio17, DEM, SLO, ASP, NDVI, POP, LC |
| *Fulica atra* | Bio2, Bio3, Bio4, Bio9, Bio10, Bio15, Bio17, Bio18, DEM, SLO, ASP, NDVI, POP, LC |
| *Grus grus* | Bio3, Bio4, Bio6, Bio8, Bio13, Bio15, Bio19, DEM, SLO, ASP, NDVI, POP, LC |
| *Grus vipio* | Bio3, Bio8, Bio15, SLO, ASP, NDVI, POP, LC |
| **Waders-Herons** | |
| *Ardea cinerea* | Bio1, Bio2, Bio3, Bio6, Bio10, Bio13, Bio15, Bio19, DEM, SLO, ASP, NDVI, POP, LC |
| *Ardeola bacchus* | Bio1, Bio2, Bio3, Bio4, Bio10, Bio13, Bio15, Bio19, DEM, SLO, ASP, NDVI, POP, LC |
| *Egretta garzetta* | Bio3, Bio6, Bio7, Bio10, Bio15, Bio16, Bio17, DEM, SLO, ASP, NDVI, POP, LC |
| *Nycticorax nycticorax* | Bio3, Bio6, Bio7, Bio8, Bio15, Bio16, Bio19, DEM, SLO, ASP, NDVI, POP, LC |
| *Platalea leucorodia* | Bio4, Bio6, Bio8, Bio13, Bio15, Bio19, DEM, SLO, ASP, NDVI, POP, LC |

**Table S4. RF index of each species**

| Ecological taxa | Species | Type of land use | | | | | |
| --- | --- | --- | --- | --- | --- | --- | --- |
|  |  | Corpland | Forest | Grassland | Water | Barren | Impervious |
| Ciconiiformes  &  Gruiformes | *Ciconia boyciana* | 0.11 | 0.19 | 11.92 | 0.55 | - | 0.12 |
|  | *Grus grus* | 0.16 | 0.05 | 0.18 | 0.57 | - | 0.15 |
|  | *Grus vipio* | 0.04 | 0.03 | - | 0.22 | - | 0.06 |
|  | *Fulica atra* | 0.47 | 0.56 | 0.65 | 2.28 | 0.78 | 0.85 |
| Shorebird | *Calidris acuminata* | 0.17 | 0.92 | - | 0.43 | 5.88 | 0.25 |
|  | *Himantopus himantopu* | 0.32 | 0.48 | 0.88 | 1.32 | 2.71 | 0.41 |
|  | *Charadrius mongolus* | 0.15 | 0.26 | 0.02 | 0.46 | 2.55 | 0.22 |
|  | *Charadrius alexandrinus* | 0.24 | 0.44 | - | 0.73 | 2.14 | 0.26 |
|  | *Calidris tenuirostris* | 0.29 | 0.81 | - | 0.94 | - | 0.21 |
|  | *Tringa stagnatilis* | 0.23 | 0.37 | - | 0.83 | 5.86 | 0.52 |
|  | *Calidris ruficollis* | 0.2 | 0.46 | - | 0.63 | 4.76 | 0.29 |
|  | *Tringa nebularia* | 0.47 | 0.46 | 4.86 | 1.05 | 3.93 | 0.52 |
|  | *Calidris ferruginea* | 0.05 | 0.27 | - | 0.28 | - | 0.09 |
|  | *Recurvirostra avosetta* | 0.24 | 0.36 | 1.15 | 1.02 | 3.25 | 0.17 |
|  | *Calidris canutus* | 0.31 | 0.79 | - | 0.32 | - | 0.23 |
|  | *Pluvialis squatarola* | 0.3 | 0.63 | - | 0.81 | 7.09 | 0.31 |
|  | *Calidris alpina* | 0.28 | 0.5 | - | 0.87 | 6.26 | 0.28 |
|  | *Numenius arquata* | 0.07 | 0.04 | - | 0.32 | 0.74 | 0.08 |
|  | *Limosa limosa* | 0.18 | 0.3 | 1.85 | 0.56 | 3.03 | 0.18 |
| Herons | *Platalea leucorodia* | 0.21 | 0.39 | 1.43 | 0.9 | - | 0.29 |
|  | *Nycticorax nycticorax* | 0.55 | 0.68 | 4.57 | 2.21 | - | 1.62 |
|  | *Egretta garzetta* | 1.05 | 0.96 | 1.76 | 3.15 | 2.95 | 2.31 |
|  | *Ardeola bacchus* | 0.41 | 0.41 | 2.07 | 1.55 | 2.88 | 0.84 |
|  | *Ardea cinerea* | 0.45 | 0.44 | 0.58 | 2.17 | 0.74 | 0.96 |
| Gulls | *Sternula albifrons* | 0.08 | 0.27 | 0.74 | 0.22 | 1.99 | 0.14 |
|  | *Sterna hirundo* | 0.14 | 0.44 | 0.14 | 0.4 | 0.58 | 0.27 |
|  | *Larus crassirostris* | 0.25 | 1.05 | - | 0.89 | 7.36 | 0.66 |
|  | *Larus* *vegae* | 0.46 | 1.37 | 2.26 | 1.59 | 3.26 | 0.75 |
|  | *Chroicocephalus ridibundus* | 0.26 | 0.19 | 0.12 | 1.34 | 0.46 | 0.41 |
|  | *Chlidonias hybrida* | 0.25 | 0.62 | 0.65 | 0.92 | 0.95 | 0.36 |
| Podicipediformes | *Tachybaptus ruficollis* | 0.55 | 0.58 | 0.85 | 2.88 | 2.03 | 1.57 |
|  | *Podiceps cristatus* | 0.29 | 0.45 | 0.27 | 2.12 | 0.51 | 0.55 |
| Anatidae | *Mareca faLCata* | 0.38 | 1.98 | - | 1.36 | - | 0.42 |
|  | *Cygnus cygnus* | 0.08 | 0.16 | - | 0.56 | - | 0.17 |
|  | *Cygnus columbianus* | 0.21 | 0.3 | - | 0.83 | - | 0.24 |
|  | *Bucephala clangula* | 0.06 | 0.58 | - | 0.44 | - | 0.19 |
|  | *Aythya ferina* | 0.21 | 0.16 | 0.24 | 1.19 | - | 0.37 |
|  | *Tadorna tadorna* | 0.15 | 0.06 | - | 1.32 | - | 0.18 |
|  | *Anser fabalis* | 0.28 | 0.7 | - | 0.86 | - | 0.2 |
|  | *Mergus merganser* | 0.24 | 0.57 | 0.24 | 1.4 | - | 0.37 |
|  | *Anser anser* | 0.12 | 0.22 | - | 0.53 | - | 0.12 |
|  | *Mergellus albellus* | 0.31 | 1.2 | - | 0.79 | - | 0.34 |
|  | *Anas zonorhyncha* | 0.49 | 0.76 | 0.67 | 2.37 | 1.6 | 0.92 |
|  | *Mareca strepera* | 0.23 | 0.38 | 0.47 | 1.27 | - | 0.3 |
|  | *Anas acuta* | 0.18 | 0.18 | 0.3 | 0.86 | - | 0.18 |
|  | *Mareca penelope* | 0.24 | 0.3 | - | 0.94 | - | 0.22 |


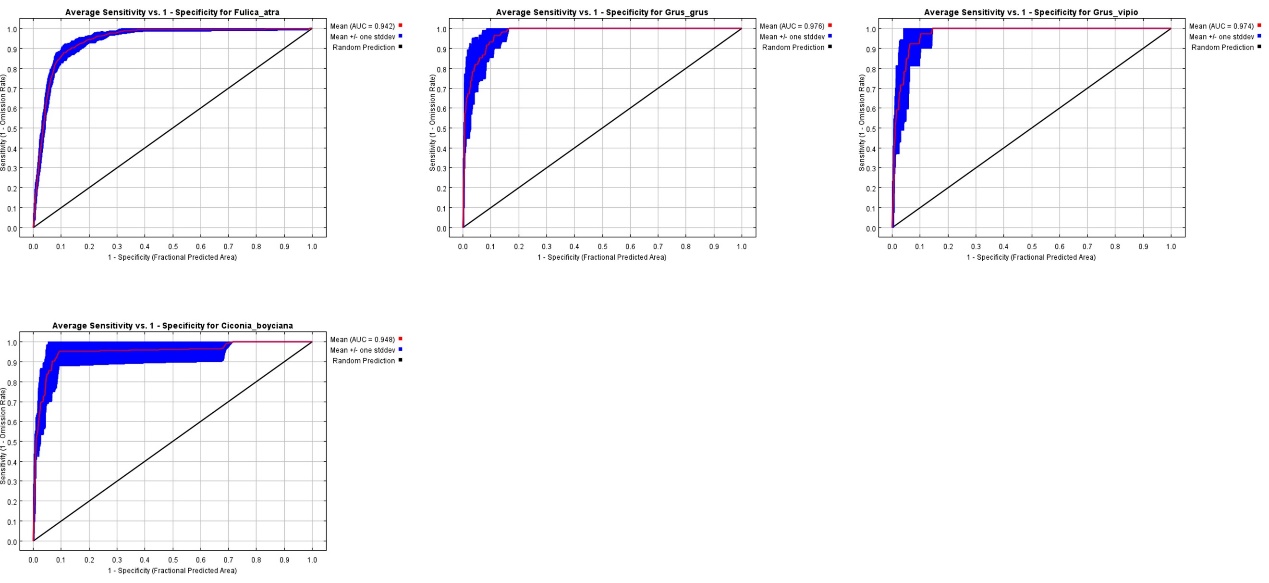


**Figure S1. MaxEnt Model Accuracy** **(AUC, dimensionless) of Ciconiiformes and Gruiformes**


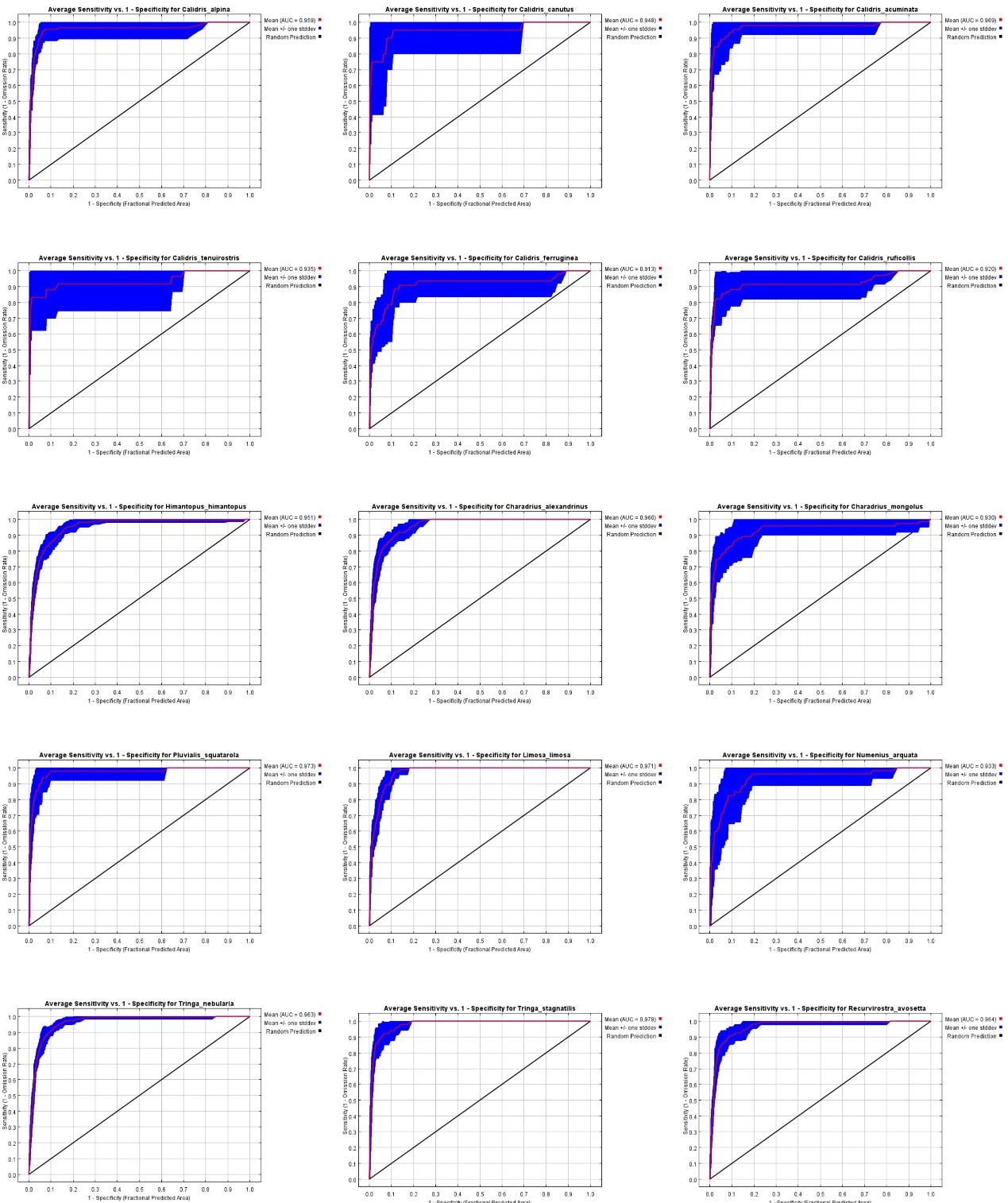


**Figure S2. MaxEnt Model Accuracy** **(AUC, dimensionless) of Shorebird**


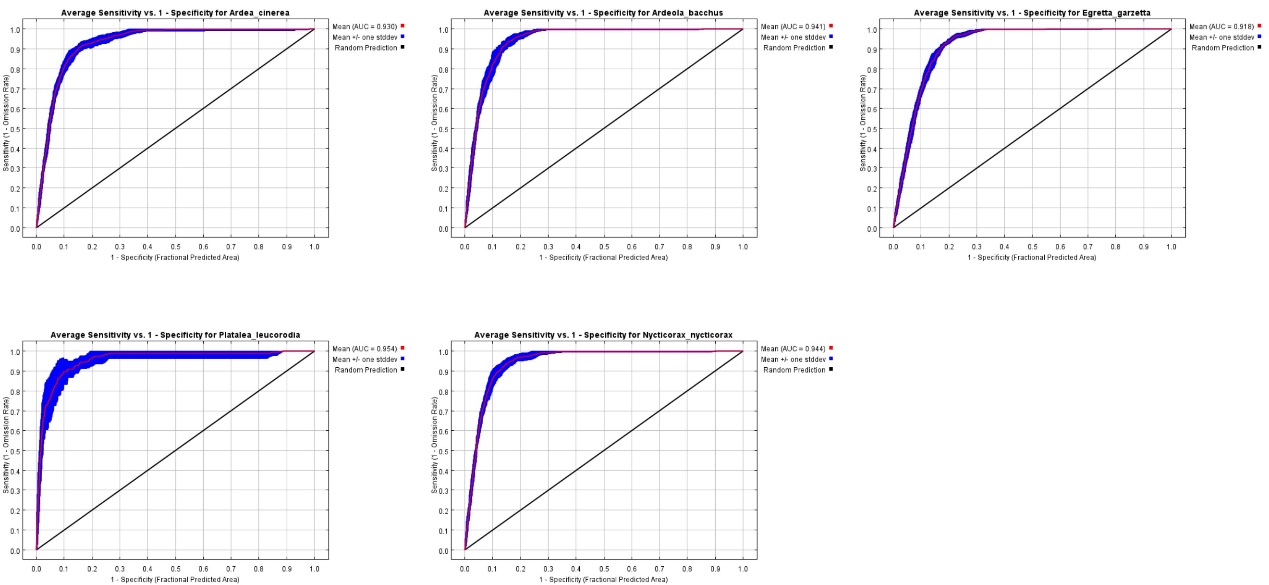


**Figure S3 MaxEnt Model Accuracy of Herons**


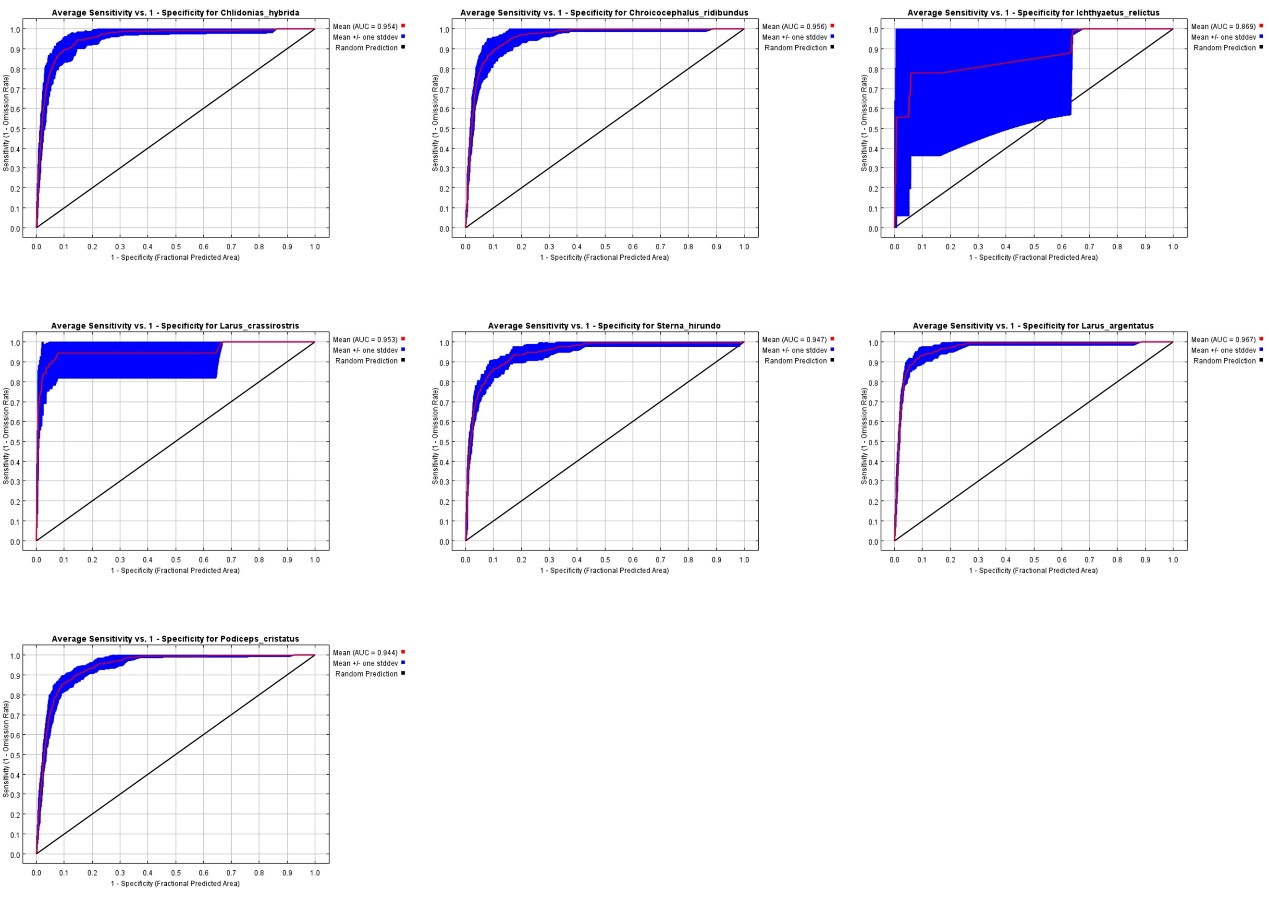


**Figure S4. MaxEnt Model Accuracy** **(AUC, dimensionless) of Gulls**


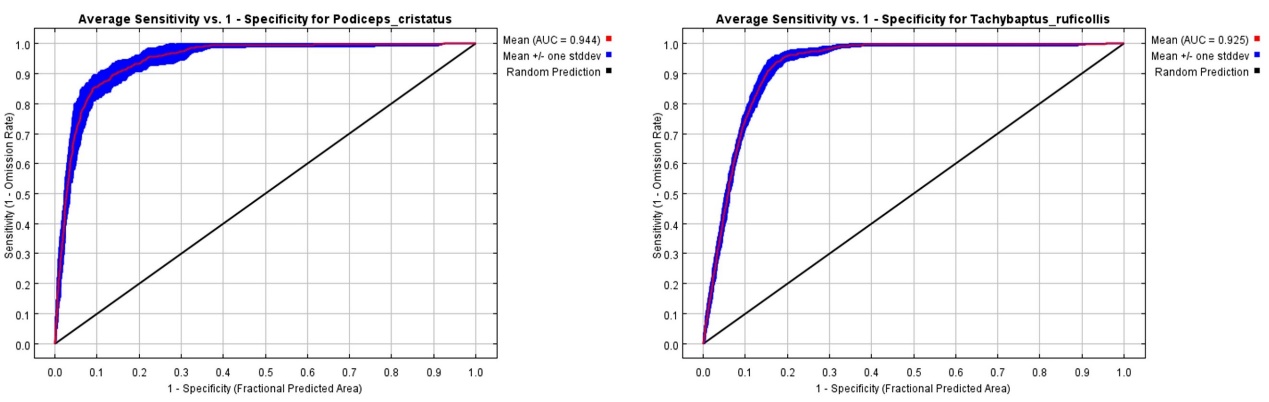


**Figure S5. MaxEnt Model Accuracy** **(AUC, dimensionless) of Podicipediformes**


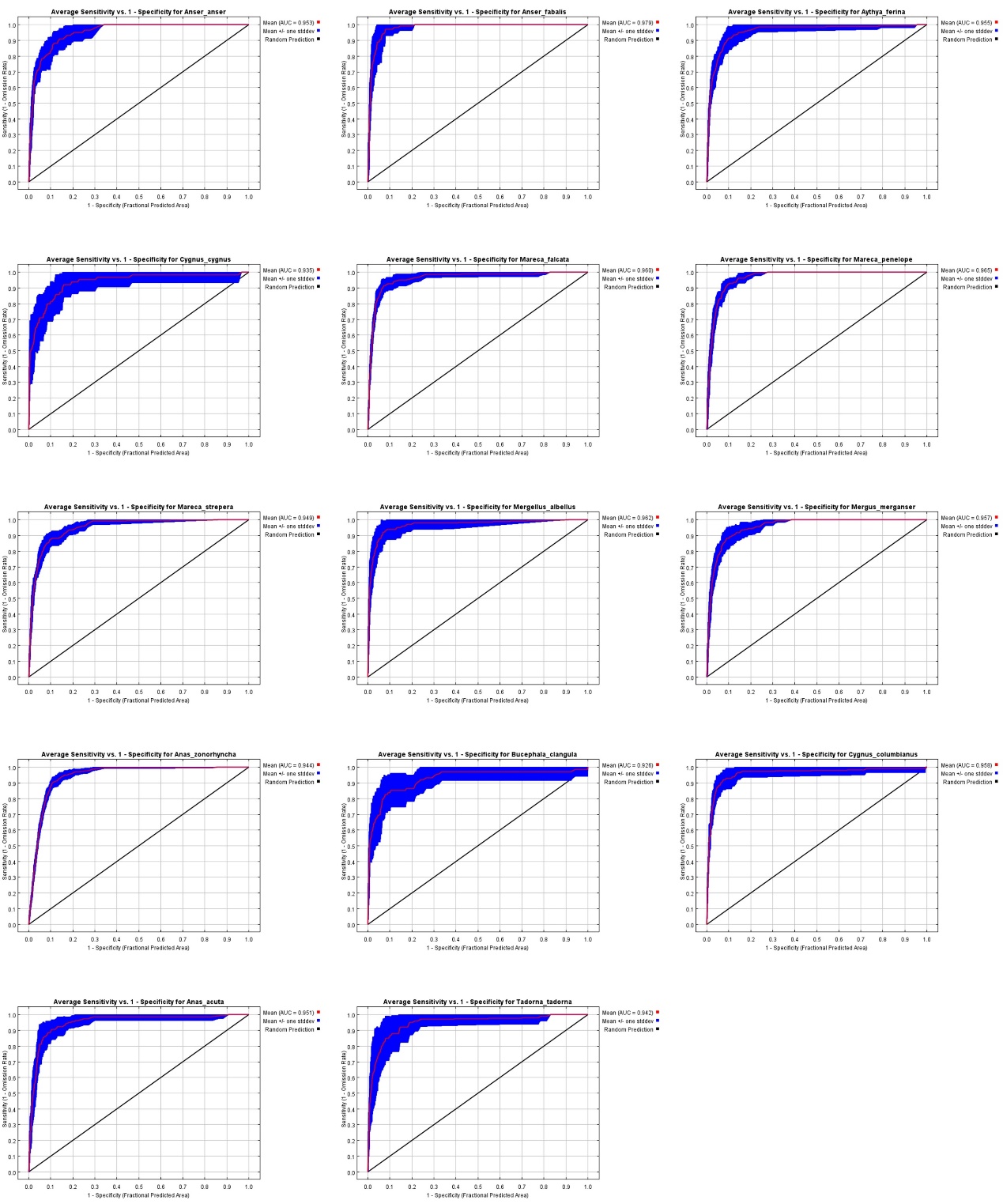


**Figure S6. MaxEnt Model Accuracy** **(AUC, dimensionless) of Anatidae**
